# Post-vaccination COVID-19: A case-control study and genomic analysis of 119 breakthrough infections in partially vaccinated individuals

**DOI:** 10.1093/cid/ciab714

**Published:** 2021-08-19

**Authors:** Ioannis Baltas, Florencia A T Boshier, Charlotte A Williams, Nadua Bayzid, Marius Cotic, José Afonso Guerra-Assunção, Dianne Irish-Tavares, Tanzina Haque, Jennifer Hart, Sunando Roy, Rachel Williams, Judith Breuer, Tabitha W Mahungu

**Affiliations:** 1 Department of Virology, Royal Free London NHS Foundation Trust, London, United Kingdom; 2 Institute of Education, University College London, London, United Kingdom; 3 Department of Infection, Immunity and Inflammation, UCL Great Ormond Street Institute of Child Health, University College London, London, United Kingdom; 4 Department of Genetics & Genomic Medicine, UCL Great Ormond Street Institute of Child Health, University College London, London, United Kingdom; 5 Department of Microbiology, Great Ormond Street Hospital for Children NHS Foundation Trust, London, United Kingdom

**Keywords:** COVID-19, Vaccination, Mortality, Genomics, Mutation

## Abstract

**Background:**

Post-vaccination infections challenge the control of the COVID-19 pandemic.

**Methods:**

We matched 119 cases of post-vaccination SARS-CoV-2 infection with BNT162b2 mRNA, or ChAdOx1 nCOV-19, to 476 unvaccinated patients with COVID-19 (Sept 2020-March 2021), according to age and sex. Differences in 60-day all-cause mortality, hospital admission, and hospital length of stay were evaluated. Phylogenetic, single nucleotide polymorphism (SNP) and minority variant allele (MVA) full genome sequencing analysis was performed.

**Results:**

116/119 cases developed COVID-19 post first vaccination dose (median 14 days, IQR 9 – 24 days). Overall, 13/119 (10∙9%) cases and 158/476 (33∙2%) controls died (p<0.001), corresponding to 4∙5 number needed to treat (NNT). Multivariably, vaccination was associated with 69∙3% (95%CI 45∙8 – 82∙6) relative risk (RR) reduction in mortality. Similar results were seen in subgroup analysis for patients with infection onset ≥14 days after first vaccination (RR reduction 65∙1%, 95%CI 27∙2 – 83∙2, NNT 4∙5), and across vaccine subgroups (BNT162b2: RR reduction 66%, 95%CI 34∙9 – 82∙2, NNT 4∙7, ChAdOx1: RR reduction 78∙4%, 95%CI 30∙4 – 93∙3, NNT 4∙1). Hospital admissions (OR 0∙80, 95%CI 0∙51 – 1∙28), and length of stay (-1∙89 days, 95%CI -4∙57 – 0∙78) were lower for cases, while Ct values were higher (30∙8 versus 28∙8, p = 0.053). B.1.1.7 was the predominant lineage in cases (100/108, 92.6%) and controls (341/446, 76.5%). Genomic analysis identified one post-vaccination case harboring the E484K vaccine escape mutation (B.1.525 lineage).

**Conclusions:**

Previous vaccination reduces mortality when B.1.1.7 is the predominant lineage. No significant lineage-specific genomic changes during phylogenetic, SNP and MVA analysis were detected.

## Introduction

Since its emergence in November 2019, SARS-CoV-2, causing coronavirus disease 2019 (COVID-19), has driven vaccine development at unprecedented speed. By January 2021, the mRNA vaccines BNT162b2 by Pfizer-BioNTech and mRNA-1273 by Moderna, as well as the vector-based vaccines ChAdOx1 nCoV-19 by Oxford-AstraZeneca and Gam-COVID-Vac (Sputnik V), produced by Russia, were already being administered under emergency use authorization in multiple countries[1-4]. Those, that rapidly deployed effective vaccination programs, like the UK or Israel, saw a dramatic decrease in cases, hospitalizations and deaths[5-8].

Despite the overwhelming success, the COVID-19 pandemic still poses a significant global public health threat. This is due to the emergence of major new variants in the United Kingdom (B.1.1.7), South Africa (B.1.351), Brazil (P.1), and India (B.1.617.2)[9]. These lineages demonstrate increased transmissibility and have raised concerns regarding reduced vaccine and treatment (monoclonal antibody) efficacy, when significant mutations are present[10-12]. Post-vaccination infections constitute a dangerous setting, where the non-sterilizing immune response may favor vaccine escape mutations[13-16]. Given the recent deployment of vaccination programs though, limited literature exists on post-vaccination COVID-19[13, 14, 16].

At the same time, there is urgent need for vaccine post-authorization studies, as the strictly controlled environment of clinical trials compromises their external validity. Real-world data is required, especially regarding clinical endpoints after infection. Authorization trials were powered to detect differences in COVID-19 cases yet studied primarily young healthy adults[1-4]. Therefore, hospital admissions and deaths were rarely recorded, despite high participant numbers. An urgent need for addressing this literature gap has been previously identified[17].

Here, we investigated 119 cases of COVID-19 infection at least one day after first vaccination with BNT162b2 or ChAdOx1, in parallel with unvaccinated age- and sex-matched patients with COVID-19 infection. We aimed to investigate the hypothesis that previous vaccination reduces mortality, admissions, and length of stay in hospital. Additionally, we compared the whole genome sequences of cases and controls to evaluate the development of vaccine-escape mutations.

## Methods

### Setting

The Royal Free Hospital (RFH) is a tertiary teaching hospital in London, with an 830 inpatient bed capacity, sharing a catchment area of 2.5 million people with two district general hospitals. It offers general and specialist services, including solid organ transplantation and renal dialysis.

The RFH collaborates with the COVID-19 Genomics UK Consortium (COG-UK), a UK publicly funded partnership of public health agencies, academic partners, diagnostic laboratories and NHS organizations, performing decentralized full-genome COVID-19 sequencing. The RFH is also a study site for the COG-UK Hospital-Onset COVID-19 Infections (HOCI) Study, a National Institute for Health Research (NIHR) Urgent Public Health-Badged Clinical Study, which aims to investigate the molecular epidemiology of SARS-CoV-2 transmission within healthcare settings. Our study was nested within the COG-UK HOCI study.

**Study design and participants**

All SARS CoV-2 first positive cases recruited into the COG-UK-HOCI study between the 30$^{th}$ of September 2020 and 15$^{th}$ of March 2021 were included. Positive patients, whose samples were not available for sequencing, were excluded. No other exclusion criteria applied. Ethical approval for the COG-UK HOCI study was provided by Research Ethics Committee (REC) 20/EE/0118. The University College London DNA Infection Bank REC waived the need for participant informed consent for this study (Ref 17/LO/1530).

Demographics, comorbidities, hospital admission details, vaccination dates and dates of death were collected for all eligible patients. Participants with documented COVID-19 vaccination at least one day before the positive sample, were assigned to the post-vaccination group (cases). Recently vaccinated patients (<14 days) were included, as preliminary evidence suggests that a mortality benefit is observed even within 14 days of vaccination [18]. This has also been described for other infections [19]. Subsequently, each case was randomly matched for age (± 3 years) and sex to a unique patient from the remaining participants (controls) in a 1:4 ratio (minimum possible ratio), in order to maximize statistical power with a predetermined number of cases [20]. The temporal mismatch of vaccinated and unvaccinated patients is further analyzed in the discussion section. This study has been reported according to the Strengthening the Reporting of Observational studies in Epidemiology (STROBE) guidelines.

**Definitions, Data sources and measurement**

COVID-19 infection was defined as the detection of SARS-CoV-2 RNA in a combined nose and throat swab using the assays described in the study protocol. Cycle threshold (Ct) was used as a proxy for infectivity. Positive samples from the Aptima assay, which reports relative light units (RLU), were excluded. Definitions of all other variables are included in the study protocol.

Comorbidities and demographics were pulled from the hospital information system using assigned ICD-10 codes. For patients without assigned codes, manual inspection of their record was performed. Dates of death and vaccination status were collected from the National Summary Care Record, the UK's national electronic summary of patients' key clinical information, sourced from their GP record". During the study period, only vaccination with BNT162b2 or ChAdOx1 was available in the UK, initiating on the 8$^{th}$ of December 2020 and 4$^{th}$ of January 2021, respectively. Investigators were blinded to patient outcomes while determining patient comorbidities and vice versa, and to the genomic analysis results.

**Outcomes**

The primary outcome was all-cause death within 60 days of the index SARS-CoV-2 positive sample. Secondary outcomes included requirement for hospital admission (within 14 days of the positive test), and length of stay during the index hospitalization, only for patients who survived their admission. Prespecified subgroup analysis was performed for patients with infections ≥14 days after vaccination and by vaccination type.

**Statistical analysis**

Statistical analysis was performed in SPSS Version 27 (IBM Corp). After matching, groups were univariably compared using Fischer's exact, Pearson's Chi squared, and Mann Whitney U test as appropriate. Multivariable Cox, logistic and linear regression were used for survival, admission and length of stay analysis respectively. Differences of 0·2 or less in confounding variables were considered acceptable, otherwise they were inserted into the multivariable model[21]. Vaccine effectiveness estimates were reported as (1 – Hazard ratio) × 100 for mortality, odds ratio for hospital admission, and difference in days for hospital length of stay.

**Genomic analysis**

During genomic analysis, investigators were blinded to clinical metadata. All samples were sequenced using either Illumina or Oxford Nanopore Technology, depending on availability. For Illumina sequencing, raw data were processed with the ARTIC NextFlow pipeline. Consensus sequences were called at 10X minimum coverage across the genome. For Nanopore sequencing, raw data in the form of fast5 files were base called using the ONT guppy high accuracy basecaller included in ONT MinkNOW for the GridION version 19.12.6 (Oxford Nanopore Technologies, Oxford, UK). Fastq files produced were demultiplexed using Porechop under the artic-ncov2019 version 1.0 pipeline. Consensus sequences at a minimum of 20X coverage were generated using the artic-ncov2019 medaka pipeline. Only samples with > 50% genome coverage were analyzed further.

A maximum likelihood tree of the consensus alignment was constructed using IQ-TREE v.2.1.2, with the GTR model and 1000 bootstrap replicates[22]. Trees were rooted on the SARS-CoV-2 reference genome MN908947.3 and visualized with ggtree. Consensus sequences were aligned using MAFFT[23]. Figures were generated in R 3.6.1 using Rstudio 1.2 with libraries dplyr, ggplot2 and ggtree[24]. Samples with >90% coverage and 10X depth were carried forward for analysis. Minority variant alleles (MVAs) were called relative to Wuhan-1 (MN908947.3), with a frequency of above 5% and with a minimum of 4 supporting reads identified at sites with a read depth of ≥ 5 using VarScan[25].

**Role of the funding source**

The funders of this study had no role during the study design, in the collection, analysis, and interpretation of data, in the writing of the report, and in the decision to submit the paper for publication.

**Results**

During the study period, 2130 patients tested positive for SARS-CoV-2 at the RFH, of which 1864 (87·5%) were enrolled in the COG-UK-HOCI study. In the final cohort, there were 119 post-vaccination cases (6.4%). The median number of days of infection detection after vaccination was 14 (IQR 9 – 24, Supplementary Table 1). Only three cases developed COVID-19 after their second dose (at days 13, 22, 56), none of whom died. Seventy-nine (66·4%) patients received BNT162b2, while forty patients (33·6%) received ChAdOx1. Detailed demographics and comorbidities are shown in Table 1. Analysis revealed an elderly (median age 79, IQR 65 – 86) and comorbid post-vaccination cohort, that was predominantly male (57·1%), and of white ethnicity (73·9%). There were high levels of chronic cardiac disease (49·6%) and diabetes (29·4%), a significant number of vulnerable patients (transplant 3·4%, immunosuppression 13·4%, and renal dialysis 6·7%), as well as young healthy adults. This population aligned with UK recommendations to prioritize vaccination in old (≥70) and clinically vulnerable patients during the study period, as well as health and social care workers.

The final cohort of 595 patients was 90% powered to detect a 13% reduction in risk of mortality, a 23% reduction in admission odds, and a 4·4-day reduction in length of stay. Notable differences in the two groups included ethnicity, chronic respiratory disease, diabetes, and chronic neurological disease, yet none was statistically significant (Table 1). Matching remained successful across all prespecified subgroups (Supplementary Tables 2 – 4). Patients with infections ≥14 days after vaccination were older (median age 82 vs 79, p = 0.49) and more likely to be immunosuppressed (N=13, 21% vs N=16, 13·4%, p = 0.20) compared to the entire cohort, while patients receiving ChAdOx1 were younger compared to patients receiving BNT162b2 (median age 75·5 vs 82, p =0.21), likely representing the delayed introduction of ChAdOx1. Observed differences were not statistically significant. The predominant lineage was B.1.1.7 (79·6%), and was more common in the post vaccination cohort (92·6% vs 76·5%, p <0.001), likely due to the temporal mismatch of cases and controls (the B.1.1.7 lineage did not become dominant in the UK until November 2020). Additional matching for sample date neutralized differences in lineage frequencies, while showing a preserved effect of vaccination on mortality (data not shown).  No other variants of concern were detected. Crude mortality in the vaccinated and unvaccinated groups was 13/119 (10·9%) and 158/476 (33·2%), respectively (p <0.001).

In a multivariable analysis, previous vaccination was associated with a 69·3% (95%CI 45·8 – 82·6) RR reduction in death before 60 days (p <0.001), and an absolute risk (AR) reduction of 22·3% (Table 2, Figure 1). This corresponds to a NNT of 4·5 vaccinations to prevent one death. Similar results were observed for patients with infection onset ≥14 days after vaccination (65·1%, 95%CI 27·2 – 83·2, AR reduction 22·2%, NNT 4·5), and across both vaccine subgroups (BNT162b2: 66%, 95%CI 34·9 – 82·2, AR reduction 21·2%, NNT 4·7, ChAdOx1: 78·4%, 95%CI 30·4 – 93·3, AR reduction 24·4%, NNT 4·1, Supplementary Figures 1-3, Supplementary Table 5). Vaccinated participants had lower odds of hospital admission (OR 0·80, 0·51 – 1·28, p = 0.36), and shorter length of stay (-1·89 days, -4·57 – 0·78, p = 0.17), but differences were not statistically significant, as predicted by power analysis (Table 2, Supplementary Tables 6,7). ChAdOx1 vaccinees demonstrated a slightly higher vaccine efficacy, and a shorter hospital stay (Table 2). Median Ct values were 30·8 (IQR 25·9 – 35·4), and 28·8 (IQR 25·3 – 33·7) p = 0.053 for cases and controls respectively (Table 1). The difference reached statistical significance in subgroup analysis for patients with infection onset ≥14 days after vaccination (30·6, IQR 26·1 – 37·1 vs 28·2 IQR 24·4 – 32·7 p = 0.005, Supplementary Table 2).

Illumina and Nanopore sequencing were used for 527/595 (88.6%) and 68/595 (11.4%) samples respectively. In total, 108/119 (90·8%) cases and 446/476 (93·7%) controls met quality criteria for further analysis (493/527, 93.5% for Ilumina and 61/68, 89.7% for Nanopore). Median genome coverage was 97.7% (IQR 94.1% - 98.8%, range 55% - 99.6%). The maximum likelihood phylogeny of sequenced samples is shown in Figure 2. We found no clustering of viral sequences from post-vaccine cases, and no cases were associated with abnormal branch length. The frequency of common SNPs (shared by more than 20% of individuals in either group) were no different between post-vaccine and controls (Figure 3). Only one potential vaccine-escape mutation, E484K, was found, in an immunocompromised patient infected with the B.1.525 lineage four days after the first dose of BNT162b2 (Supplementary Table 8). The mean number of MVAs was not statistically different between the two groups (cases = 2.6, controls = 2.2, p=0.17). GISAID accession numbers for all sequenced samples with ≥90% coverage are included in the appendix.

**Discussion**

This study describes a cohort of BNT162b2 or ChAdOx1 vaccinated multimorbid patients developing COVID-19 predominantly from the B.1.1.7 lineage post first vaccination. In this cohort, one life was saved every four to five vaccinations. Genomic analysis identified one patient with an escape mutation (E484K; B.1.525 lineage) who developed COVID-19 four days post vaccination.

We describe a real-world setting, where a mass vaccination campaign was rolled out during a pandemic. We therefore acknowledge that a proportion of cases would have been infected before vaccination, therefore were not strictly post-vaccination infections[26]. Yet, this is more a theoretical concern, rather than a factor that confounds our results. Our data strongly suggests that these patients have a much more favorable outcome to their matched counterparts, whether they had "preventative" (pre-infection) or "therapeutic" (post infection) vaccination.

Our data indirectly questions whether pauci-symptomatic/asymptomatic patients should be offered vaccination, as we have demonstrated a survival benefit in patients developing COVID-19 shortly after being vaccinated. Further studies are required to explore this hypothesis. Figure 1 suggests that the mortality benefit from vaccination occurs immediately after COVID-19 infection, as Kaplan Meier curves diverge early, suggesting rapid protection. Our data corroborates the latest PHE vaccine effectiveness report, suggesting reduced risk of death at population level being measurable within the first two-week period after vaccination, while reduced risk of hospitalization is only observed after the initial two weeks[7]. A nationwide study from Israel also demonstrated that reduction in deaths is achieved earlier than other outcomes[5]. A cluster study in USA care homes showed that protection from a composite outcome of death or hospitalization was observed within the first week after vaccination[27]. Evidence is suggestive that a substantial mortality benefit is obtainable early after first vaccination, while avoidance of hospitalizations or cases might require additional time. Our study strongly supports the practice of delaying second vaccination to provide a greater degree of protection at a population level, in settings where vaccines are limited.

We did not demonstrate a statistically significant reduction in hospitalizations and length of stay in hospital after vaccination in contrast with previous studies,[7, 8] as our cohort was clearly underpowered for these outcomes. Additionally, our population mainly consisted of multimorbid patients presenting to the emergency department with COVID-19 symptoms, therefore having a high baseline probability of admission, compared to studies looking at community patients. Our cohort also included patients within the first 14 days of vaccination, during which literature suggests that there is still minimal effect from vaccination[7]. This might have diluted our findings.

Our study is subject to multiple limitations. Our results only apply to the vaccines studied and to B.1.1.7 lineage. Our study population might not be representative of most institutions, given the high proportion of patients with rare conditions. Despite matching, we cannot completely eliminate residual confounding, especially with regards to the fact that our cases had been offered and accepted vaccination, while our controls might have not. We elected to match for age and gender only to allow a bigger sample size. Some non-statistically significant differences were observed.

Additional matching would have put us at risk of overmatching, which can actually increase confounding[20].

We did not adjust for baseline COVID-19 serology or for previous history of infection, as this was not an interventional study, nor was the latter consistently documented. Natural infection is associated with a good degree of protection; therefore we would not expect many of our patients to have had previous episodes[28]. Additionally, it is unclear whether previous infection would increase or decrease vaccination uptake, therefore we cannot comment on the direction of potential confounding. Finally, a proportion of our cohort (between the 30th of September and the 7th of December) did not have the opportunity to be vaccinated, and therefore develop post-vaccine infection. They were also less likely to be infected with the B.1.1.7 lineage. We decided to include them as controls due to power considerations, and to aid the genomic analysis. Given that breakthroughs in COVID-19 therapeutics preceded our study period, and management of COVID-19 patients in the RFH did not substantially change throughout this time, we consider them an appropriate control for our study, with comparable background risk of mortality.

In summary, this study serves to inform clinicians and policy makers that vaccination with BNT162b2 or ChAdOx1 constitutes a powerful tool against the B.1.1.7 variant of concern. Although our results were reassuring for viral genomic changes post vaccination, further surveillance of the impact of vaccine escape mutations in vaccinees is required.

## NOTES

### Contributors

IB conceived this study, determined study design and did the literature search. IB and FTB wrote the study's draft protocol and methodology, which was edited and approved by all authors. IB, TWM, TH, DIT, and JH investigated, curated and analyzed the clinical data, FTB and JB the genomic data. RW, SR, JAG-A, CAW, NB, MC sequenced the clinical samples. The study was supervised by TWM and JB. JB acquired funding. Underlying clinical and genomic data was verified by IB, TWM and FTB, JB respectively. IB wrote the original draft. All authors edited the manuscript for important intellectual content.  All authors had full access to the deidentified and aggregated data in the study, approved the final version of the manuscript and are responsible for the decision to submit for publication.

### Acknowledgements

We thank all study participants. We thank Darren Jones, for assisting with extraction of the clinical data. We thank Eirini Koutoumanou, for assisting with statistical analysis. This study was made possible through the COG-UK Consortium, supported by funding from the Medical Research Council (MRC) part of UK Research & Innovation (UKRI), the National Institute of Health Research (NIHR) and Genome Research Limited, operating as the Wellcome Sanger Institute. We thank the John Black Charitable Foundation.

### Funding

This work was supported by funding awarded to JB for the conduction of the COG-UK Hospital-Onset COVID-19 Infections (HOCI) Wellcome Trust Study through grants from UK Research and Innovation (UKRI); the Wellcome Trust; and the John Black Charitable Foundation.

**Declarations of interest**

IB reports personal fees from Shionogi B.V. for presentations in medical conferences on the topic of Gram-negative infection management outside the submitted work. All other authors have no conflicts of interest to declare.

## Table 1: Study participants

| Variables | Cases (N =119) | Controls (N = 476) | p |
|---|---|---|---|
| Age | 79 (65 – 86) | 79 (66 – 86) | 0.70 |
| <30 | 2 (1·7%) | 9 (1·9%) | 1 |
| 30 - 39 | 3 (2·5%) | 11 (2·3%) | |
| 40 - 49 | 2 (1·7%) | 10 (2·1%) | |
| 50 - 59 | 15 (12·6%) | 59 (12·4%) | |
| 60 - 69 | 11 (9·2%) | 47 (9·9%) | |
| 70 - 79 | 27 (22·7%) | 116 (24·3%) | |
| >80 | 59 (49·6%) | 224 (47·1%) | |
| Sex | | | |
| Male | 68 (57·1%) | 272 (57·1%) | 1 |
| Female | 51 (42·9%) | 204 (42·9%) | |
| Ethnicity | | | |
| White | 88 (73·9%) | 323 (67·9%) | 0.17 |
| Asian | 25 (21·1%) | 104 (21·8%) | |
| Black | 6 (5%) | 33 (6·9%) | |
| Mixed/Other | 0 (0%) | 16 (3·4%) | |
| MID Quartile[a] | | | |
| 1st | 20 (16·8%) | 106 (22·3%) | 0.51 |
| 2nd | 29 (24·4%) | 105 (22%) | |
| 3rd | 37 (31·1%) | 127 (26·7%) | |
| 4th | 33 (27·7%) | 138 (29%) | |
| Nursing/Care home resident | 6 (5%) | 22 (4·6%) | 0.81 |
| Lineage[b] | | | |
| B.1.1.7 | 100 (84%) | 341 (71·6%) | <0.001 |
| Other | 8 (6·7%) | 105 (22·1%) | |
| Low quality | 11 (9·3%) | 30 (6·3%) | |
| Days from vaccination[c] | 14 (9 – 24) | N/A | |
| Pregnancy | 0 (0%) | 9 (1·9%) | 0.22 |
| Chronic renal disease | 16 (13·4%) | 62 (13%) | 0.88 |
| Immunosuppression | 16 (13·4%) | 59 (12·4%) | 0.76 |
| Obesity | 5 (4·2%) | 31 (6·5%) | 0.52 |
| Transplant | 4 (3·4%) | 11 (2·3%) | 0.52 |
| Asplenia | 0 (0%) | 2 (0·4%) | 1 |
| HIV | 0 (0%) | 1 (0·2%) | 1 |
| Chronic respiratory disease | 14 (11·8%) | 90 (18·9%) | 0.08 |
| Asthma | 8 (6·7%) | 45 (9·5%) | 0.47 |
| Chronic cardiac disease | 59 (49·6%) | 257 (54%) | 0.41 |
| Renal dialysis | 8 (6·7%) | 31 (6·5%) | 1 |
| Chronic liver disease | 8 (6·7%) | 38 (8%) | 0.85 |
| Diabetes | 35 (29·4%) | 171 (35·9%) | 0.20 |
| Chronic neurological disease | 19 (16%) | 110 (23·1%) | 0.11 |
| Active solid organ malignancy | 15 (12·6%) | 50 (10·5%) | 0.51 |
| Haematological disease | 9 (7·6%) | 29 (6·1%) | 0.53 |
| Rheumatological disease | 13 (10·9%) | 40 (8·4%) | 0.37 |
| Dementia | 18 (15·1%) | 83 (17·4%) | 0.59 |
| Ct value[d] | 30·8 (25·9 – 35·4) | 28·8 (25·3 – 33·7) | 0.053 |

| | | | |
|---|---|---|---|
| Admission to hospital | 86 (72·3%) | 371 (77·9%) | 0.22 |
| Length of stay in hospital[e] | 6·5 (3·75 – 11·25) | 8 (4 – 16) | 0.07 |
| Death | 13 (10·9%) | 158 (33·2%) | <0.001 |

Continuous variables are presented as median (IQR), categorical variables as N (%).[a]The first quartile represents the least deprived participants. [b]Other includes wild-type COVID-19, or lineages that have not been characterized as variants of concern. Not all samples met sequencing quality criteria. [c]Indicates days since first vaccination. [d]N = 112 for cases and N = 399 for controls, excludes samples tested in the Aptima platform.[e]N = 78 for cases and N = 259 for controls, only includes patient that were admitted and survived their admission.

**Table 2: Vaccine Effectiveness[a]**

| | Death | p | Admission | p | Length of stay | p |
|---|---|---|---|---|---|---|
| Entire cohort[b] N =119 | 69·3 (45·8-82·6) | <0.001 | 0·80 (0·51-1·28) | 0.36 | -1·89 (-4·57 - 0·78) | 0.17 |
| Vaccination≥14 days[c] N= 62 (3) | 65·1 (27·2-83·2) | 0.005 | 0·57 (0·30-1·09) | 0.09 | -2·36 (-5·74-1·02) | 0.17 |
| BNT162b2[d] N = 79 | 66·0 (34·9-82·2) | 0.001 | 0·75 (0·43-1·31) | 0.31 | -0·92 (-3·83-1·98) | 0.53 |
| ChAdOx1[e] N = 40 | 78·4 (30·4-93·3) | 0.01 | 0·80 (0·35 - 1·81) | 0.59 | -3·98 (-9·45-1·58) | 0.15 |

Table 1: [a]Vaccine effectiveness estimates are reported as (1 – Hazard ratio) × 100 (95%CI) for death, odds ratio (95%CI) for admission to hospital, and difference in days (95%CI) for length of stay in hospital. [b]Adjusted for ethnicity, chronic respiratory disease, diabetes, chronic neurological disease, [c]Adjusted for immunosuppression, chronic respiratory disease, renal dialysis, chronic neurological disease, active solid organ malignancy, rheumatological disease, [d]Adjusted for chronic neurological disease. [e]Adjusted for chronic respiratory disease.

FIGURE LEGENDS

Figure 1: Cumulative incidence curves (1 minus hazard ratio) for all-cause death before 60 days for study cases and controls, starting from the day of the index positive COVID-19 sample. Numbers at risk at each time point and numbers censored are also shown.

Figure 2: Maximum likelihood phylogeny of successfully sequenced samples using a generalized time-reversible model. The tree is routed on the reference strain MN908947.3, and each branch supported by 1000 bootstraps.

Figure 3: Single nucleotide polymorphisms (SNPs) frequency from successfully sequenced samples from cases and controls across the annotated SARS-CoV-2 MN908947.3 references genome.

Figure 1

Accepted Ma

Figure 2

## Supplementary Material

ciab714_suppl_Supplementary_Tables_Figures

ciab714_suppl_Supplementary_Appendix

ciab714_suppl_Supplementary_Materials

