# Post-Vaccination Coronavirus Disease 2019: A Case-Control Study and Genomic Analysis of 119 Breakthrough Infections in Partially Vaccinated Individuals^[Author-notes ciab714-FM1]^

**DOI:** 10.1093/cid/ciab714

**Published:** 2021-08-19

**Authors:** Ioannis Baltas, Florencia A T Boshier, Charlotte A Williams, Nadua Bayzid, Marius Cotic, José Afonso Guerra-Assunção, Dianne Irish-Tavares, Tanzina Haque, Jennifer Hart, Sunando Roy, Rachel Williams, Judith Breuer, Tabitha W Mahungu

**Affiliations:** Department of Virology, Royal Free London NHS Foundation Trust, London, United Kingdom; Institute of Education, University College London, London, United Kingdom; Department of Infection, Immunity and Inflammation, UCL Great Ormond Street Institute of Child Health, University College London, London, United Kingdom; Department of Genetics & Genomic Medicine, UCL Great Ormond Street Institute of Child Health, University College London, London, United Kingdom; Department of Genetics & Genomic Medicine, UCL Great Ormond Street Institute of Child Health, University College London, London, United Kingdom; Department of Genetics & Genomic Medicine, UCL Great Ormond Street Institute of Child Health, University College London, London, United Kingdom; Department of Genetics & Genomic Medicine, UCL Great Ormond Street Institute of Child Health, University College London, London, United Kingdom; Department of Virology, Royal Free London NHS Foundation Trust, London, United Kingdom; Department of Virology, Royal Free London NHS Foundation Trust, London, United Kingdom; Department of Virology, Royal Free London NHS Foundation Trust, London, United Kingdom; Department of Infection, Immunity and Inflammation, UCL Great Ormond Street Institute of Child Health, University College London, London, United Kingdom; Department of Genetics & Genomic Medicine, UCL Great Ormond Street Institute of Child Health, University College London, London, United Kingdom; Department of Infection, Immunity and Inflammation, UCL Great Ormond Street Institute of Child Health, University College London, London, United Kingdom; Department of Microbiology, Great Ormond Street Hospital for Children NHS Foundation Trust, London, United Kingdom; Department of Genetics & Genomic Medicine, UCL Great Ormond Street Institute of Child Health, University College London, London, United Kingdom

**Keywords:** COVID-19, vaccination, mortality, genomics, mutation

## Abstract

**Background:**

Post-vaccination infections challenge the control of the coronavirus disease 2019 (COVID-19) pandemic.

**Methods:**

We matched 119 cases of post-vaccination severe acute respiratory syndrome coronavirus 2 infection with BNT162b2 mRNA or ChAdOx1 nCOV-19 to 476 unvaccinated patients with COVID-19 (September 2020–March 2021) according to age and sex. Differences in 60-day all-cause mortality, hospital admission, and hospital length of stay were evaluated. Phylogenetic, single-nucleotide polymorphism (SNP), and minority variant allele (MVA) full-genome sequencing analysis was performed.

**Results:**

Overall, 116 of 119 cases developed COVID-19 post–first vaccination dose (median, 14 days). Thirteen of 119 (10.9%) cases and 158 of 476 (33.2%) controls died (*P* < .001), corresponding to the 4.5 number needed to treat (NNT). Multivariably, vaccination was associated with a 69.3% (95% confidence interval [CI]: 45.8 to 82.6) relative risk (RR) reduction in mortality. Similar results were seen in subgroup analysis for patients with infection onset ≥14 days after first vaccination and across vaccine subgroups. Hospital admissions (odds ratio, 0.80; 95% CI: .51 to 1.28) and length of stay (–1.89 days; 95% CI: –4.57 to 0.78) were lower for cases, while cycle threshold values were higher (30.8 vs 28.8, *P* = .053). B.1.1.7 was the predominant lineage in cases (100 of 108, 92.6%) and controls (341 of 446, 76.5%). Genomic analysis identified 1 post-vaccination case that harbored the E484K vaccine-escape mutation (B.1.525 lineage).

**Conclusions:**

Previous vaccination reduces mortality when B.1.1.7 is the predominant lineage. No significant lineage-specific genomic changes during phylogenetic, SNP, and MVA analysis were detected.

## Teaser

Previous vaccination with either BNT162b2 or ChAdOx1 reduces 60-day mortality from coronavirus disease 2019 from the B0.1.1.7 lineage by 69.3%. Breakthrough infections are not primarily driven by viral genomic mutations.

Since its emergence in November 2019, severe acute respiratory syndrome coronavirus 2 (SARS-CoV-2), which causes coronavirus disease 2019 (COVID-19), has driven vaccine development at unprecedented speed. By January 2021, the mRNA vaccines BNT162b2 by Pfizer-BioNTech and mRNA-1273 by Moderna, as well as the vector-based vaccines ChAdOx1 nCoV-19 by Oxford-AstraZeneca and Gam-COVID-Vac (Sputnik V), produced by Russia, were already being administered under emergency use authorization in multiple countries [1–4]. Countries that rapidly deployed effective vaccination programs, such as the United Kingdom and Israel, saw a dramatic decrease in cases, hospitalizations, and deaths [5–8].

Despite the overwhelming success, the COVID-19 pandemic still poses a significant global public health threat. This is due to the emergence of major new variants in the United Kingdom (B.1.1.7), South Africa (B.1.351), Brazil (P.1), and India (B.1.617.2) [9]. These lineages demonstrate increased transmissibility and have raised concerns regarding reduced vaccine and treatment (monoclonal antibody) efficacy when significant mutations are present [10–12]. Post-vaccination infections constitute a dangerous setting, where the nonsterilizing immune response may favor vaccine-escape mutations [13–16]. Given the recent deployment of vaccination programs, limited literature exists on post-vaccination COVID-19 [13, 14, 16].

At the same time, there is urgent need for vaccine post-authorization studies, as the strictly controlled environment of clinical trials compromises their external validity. Real-world data are required, especially regarding clinical end points after infection. Authorization trials were powered to detect differences in COVID-19 cases yet studied primarily young, healthy adults [1–4]. Therefore, hospital admissions and deaths were rarely recorded, despite high participant numbers. An urgent need to address this literature gap has been previously identified [17].

Here, we investigated 119 cases of COVID-19 infection at least 1 day after first vaccination with BNT162b2 or ChAdOx1 in parallel with unvaccinated age- and sex-matched patients with COVID-19 infection. We aimed to investigate the hypothesis that previous vaccination reduces mortality, admissions, and length of hospital stay. Additionally, we compared the whole-genome sequences of cases and controls to evaluate the development of vaccine-escape mutations.

## METHODS

### Setting

The Royal Free Hospital (RFH) is a tertiary teaching hospital in London, with an 830 inpatient bed capacity, sharing a catchment area of 2.5 million people with 2 district general hospitals. It offers general and specialist services, including solid organ transplantation and renal dialysis.

The RFH collaborates with the COVID-19 Genomics UK Consortium (COG-UK), a UK publicly funded partnership of public health agencies, academic partners, diagnostic laboratories, and National Health Service (NHS) organizations, to perform decentralized full-genome SARS-CoV-2 sequencing. The RFH is also a study site for the COG-UK Hospital-Onset COVID-19 Infections (HOCI) Study, a National Institute for Health Research Urgent Public Health-Badged Clinical Study that aims to investigate the molecular epidemiology of SARS-CoV-2 transmission within healthcare settings. Our study was nested within the COG-UK HOCI study.

### Study Design and Participants

All SARS-CoV-2 first positive cases recruited into the COG-UK-HOCI study between 30 September 2020 and 15 March 2021 were included. Positive patients whose samples were not available for sequencing were excluded. No other exclusion criteria applied. Ethical approval for the COG-UK HOCI study was provided by the Research Ethics Committee (REC) 20/EE/0118. The University College London DNA Infection Bank REC waived the need for participant informed consent for this study (Ref 17/LO/1530).

Demographics, comorbidities, hospital admission details, vaccination dates, and dates of death were collected for all eligible patients. Participants with documented COVID-19 vaccination at least 1 day before the positive sample were assigned to the post-vaccination group (cases). Recently vaccinated patients (<14 days) were included, as preliminary evidence suggests that a mortality benefit is observed even within 14 days of vaccination [7]. This has also been described for other infections [18]. Subsequently, each case was randomly matched for age (±3 years) and sex to a unique patient from the remaining participants (controls) in a 1:4 ratio (minimum possible ratio) in order to maximize statistical power with a predetermined number of cases [19]. The temporal mismatch of vaccinated and unvaccinated patients is further analyzed in the discussion section. This study has been reported according to the STrengthening the Reporting of OBservational studies in Epidemiology guidelines.

### Definitions, Data Sources, and Measurement

COVID-19 infection was defined as the detection of SARS-CoV-2 RNA in a combined nose and throat swab using the assays described in the study protocol. Cycle threshold (Ct) was used as a proxy for infectivity. Positive samples from the Aptima assay, which reports relative light units, were excluded. Definitions of all other variables are included in the study protocol (Supplementary Materials).

Comorbidities and demographics were pulled from the hospital information system using assigned *International Classification of Diseases, Tenth Revision, Clinical Modification*, codes. For patients without assigned codes, manual inspection of their record was performed. Dates of death and vaccination status were collected from the National Summary Care Record, the United Kingdom’s national electronic summary of patients’ key clinical information, sourced from their general practise record. During the study period, only vaccination with BNT162b2 or ChAdOx1 was available in the United Kingdom, initiating on 8 December 2020 and 4 January 2021, respectively. Investigators were blinded to patient outcomes while determining patient comorbidities and vice versa and to the genomic analysis results.

### Outcomes

The primary outcome was all-cause death within 60 days of the index SARS-CoV-2–positive sample. Secondary outcomes included requirement for hospital admission (within 14 days of the positive test) and length of stay during the index hospitalization, only for patients who survived their admission. Prespecified subgroup analysis was performed for patients with infections ≥14 days after vaccination and by vaccination type.

### Statistical Analysis

Statistical analysis was performed using SPSS version 27 (IBM Corp). After matching, groups were univariably compared using the Fischer exact, Pearson *χ*^2^, or Mann-Whitney *U* test as appropriate. Multivariable Cox, logistic, and linear regression were used for survival, admission, and length of stay analysis, respectively. Differences of 0.2 or less in confounding variables were considered acceptable, otherwise they were inserted into the multivariable model [20]. Vaccine effectiveness estimates were reported as (1 – hazard ratio) × 100 for mortality, odds ratio for hospital admission, and difference in days for hospital length of stay.

### Genomic Analysis

During genomic analysis, investigators were blinded to clinical metadata. All samples were sequenced using either Illumina or Oxford Nanopore Technology, depending on availability. For Illumina sequencing, raw data were processed with the ARTIC NextFlow pipeline. Consensus sequences were called at 10× minimum coverage across the genome. For Nanopore sequencing, raw data in the form of fast5 files were base-called using the ONT guppy high-accuracy basecaller included in ONT MinkNOW for the GridION version 19.12.6 (Oxford Nanopore Technologies, Oxford, UK). Fastq files produced were demultiplexed using Porechop under the artic-ncov2019 version 1.0 pipeline. Consensus sequences at a minimum of 20× coverage were generated using the artic-ncov2019 medaka pipeline. Only samples with >50% genome coverage were analyzed further.

A maximum likelihood tree of the consensus alignment was constructed using IQ-TREE v0.2.1.2, with the GTR model and 1000 bootstrap replicates [21]. Trees were rooted on the SARS-CoV-2 reference genome MN908947.3 and visualized with ggtree. Consensus sequences were aligned using MAFFT [22]. Figures were generated in R 3.6.1 using Rstudio 1.2 with libraries dplyr, ggplot2, and ggtree [23]. Samples with >90% coverage and 10× depth were carried forward for analysis. Minority variant alleles (MVAs) were called relative to Wuhan-1 (MN908947.3), with a frequency of above 5% and with a minimum of 4 supporting reads identified at sites with a read depth of ≥5 using VarScan [24].

## RESULTS

During the study period, 2130 patients tested positive for SARS-CoV-2 at the RFH, of which 1864 (87.5%) were enrolled in the COG-UK-HOCI study. In the final cohort, there were 119 post-vaccination cases (6.4%). The median number of days of infection detection after vaccination was 14 (interquartile range [IQR], 9–24; Supplementary Table 1). Only 3 cases developed COVID-19 after their second dose (at days 13, 22, and 56), none of whom died. Seventy-nine (66.4%) patients received BNT162b2, while 40 patients (33.6%) received ChAdOx1. Detailed demographics and comorbidities are shown in Table 1. Analysis revealed an elderly (median age, 79 years; IQR, 65–86) and comorbid post-vaccination cohort that was predominantly male (57.1%) and of White ethnicity (73.9%). There were high levels of chronic cardiac disease (49.6%) and diabetes (29.4%), a significant number of vulnerable patients (transplant, 3.4%; immunosuppression, 13.4%; and renal dialysis, 6.7%), as well as young, healthy adults. This population aligned with UK recommendations to prioritize vaccination in elderly (≥70) and clinically vulnerable patients during the study period, as well as health and social care workers.

**Table 1.** Study Participants

Variable	Cases (N = 119)	Controls (N = 476)	P Value
Age, years	79 (65–86)	79 (66–86)	.70
<30	2 (1.7%)	9 (1.9%)	1
30–39	3 (2.5%)	11 (2.3%)	
40–49	2 (1.7%)	10 (2.1%)	
50–59	15 (12.6%)	59 (12.4%)	
60–69	11 (9.2%)	47 (9.9%)	
70–79	27 (22.7%)	116 (24.3%)	
>80	59 (49.6%)	224 (47.1%)	
Sex			
Male	68 (57.1%)	272 (57.1%)	1
Female	51 (42.9%)	204 (42.9%)	
Ethnicity			
White	88 (73.9%)	323 (67.9%)	.17
Asian	25 (21.1%)	104 (21.8%)	
Black	6 (5%)	33 (6.9%)	
Mixed/Other	0 (0%)	16 (3.4%)	
Multiple index of deprivation (MID) quartile^a^			
1st	20 (16.8%)	106 (22.3%)	.51
2nd	29 (24.4%)	105 (22%)	
3rd	37 (31.1%)	127 (26.7%)	
4th	33 (27.7%)	138 (29%)	
Nursing/Care home resident	6 (5%)	22 (4.6%)	.81
Lineage^b^			
B.1.1.7	100 (84%)	341 (71.6%)	<.001
Other	8 (6.7%)	105 (22.1%)	
Low quality	11 (9.3%)	30 (6.3%)	
Days from vaccination^c^	14 (9–24)	N/A	
Comorbidities			
Pregnancy	0 (0%)	9 (1.9%)	.22
Chronic renal disease	16 (13.4%)	62 (13%)	.88
Immunosuppression	16 (13.4%)	59 (12.4%)	.76
Obesity	5 (4.2%)	31 (6.5%)	.52
Transplant	4 (3.4%)	11 (2.3%)	.52
Asplenia	0 (0%)	2 (0.4%)	1
Human immunodeficiency virus	0 (0%)	1 (0.2%)	1
Chronic respiratory disease	14 (11.8%)	90 (18.9%)	.08
Asthma	8 (6.7%)	45 (9.5%)	.47
Chronic cardiac disease	59 (49.6%)	257 (54%)	.41
Renal dialysis	8 (6.7%)	31 (6.5%)	1
Chronic liver disease	8 (6.7%)	38 (8%)	.85
Diabetes	35 (29.4%)	171 (35.9%)	.20
Chronic neurological disease	19 (16%)	110 (23.1%)	.11
Active solid organ malignancy	15 (12.6%)	50 (10.5%)	.51
Hematological disease	9 (7.6%)	29 (6.1%)	.53
Rheumatological disease	13 (10.9%)	40 (8.4%)	.37
Dementia	18 (15.1%)	83 (17.4%)	.59
Cycle threshold value^d^	30.8 (25.9–35.4)	28.8 (25.3–33.7)	.053
Admission to hospital	86 (72.3%)	371 (77.9%)	.22
Length of stay in hospital^e^	6.5 (3.75–11.25)	8 (4–16)	.07
Death	13 (10.9%)	158 (33.2%)	<.001

Continuous variables are presented as median (interquartile range), categorical variables as N (%).

^a^The first quartile represents the least deprived participants.

^b^Other includes wild-type coronavirus disease 2019 or lineages that have not been characterized as variants of concern. Not all samples met sequencing quality criteria.

^c^Indicates days since first vaccination.

^d^N = 112 for cases and N = 399 for controls, excludes samples tested in the Aptima platform.

^e^N = 78 for cases and N = 259 for controls, only includes patient who were admitted and survived their admission.

The final cohort of 595 patients was 90% powered to detect a 13% reduction in risk of mortality, a 23% reduction in admission odds, and a 4.4-day reduction in length of stay. Notable differences in the 2 groups included ethnicity, chronic respiratory disease, diabetes, and chronic neurological disease, yet none were statistically significant (Table 1). Matching remained successful across all prespecified subgroups (Supplementary Tables 2–4). Patients with infections ≥14 days after vaccination were older (median age, 82 vs 79 years; *P* = .49) and more likely to be immunosuppressed (N = 13, 21% vs N = 16, 13.4%; *P* = .20) compared with the entire cohort, while patients who received ChAdOx1 were younger compared with patients who received BNT162b2 (median age, 75.5 vs 82 years; *P* = .21), likely representing the delayed introduction of ChAdOx1. Observed differences were not statistically significant. The predominant lineage was B.1.1.7 (79.6%) and was more common in the post-vaccination cohort (92.6% vs 76.5%, *P* < .001), likely due to the temporal mismatch of cases and controls (the B.1.1.7 lineage did not become dominant in the United Kingdom until November 2020). Additional matching for sample date neutralized differences in lineage frequencies, while showing a preserved effect of vaccination on mortality (data not shown). No other variants of concern were detected. Crude mortality in the vaccinated and unvaccinated groups was 13 of 119 (10.9%) and 158 of 476 (33.2%), respectively (*P* < .001).

**Figure 1.**
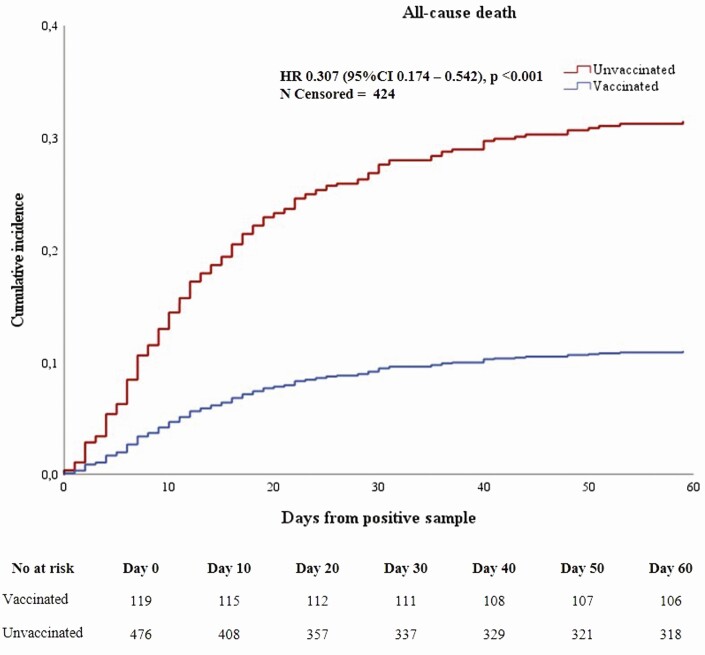
Cumulative incidence curves (1 minus hazard ratio) for all-cause death before 60 days for study cases and controls, starting from the day of the index positive coronavirus disease 2019 sample. Numbers at risk at each time point and numbers censored are also shown.

In a multivariable analysis, previous vaccination was associated with a 69.3% (95% confidence interval [CI]: 45.8 to 82.6) relative risk reduction in death before 60 days (*P* < .001) and an absolute risk (AR) reduction of 22.3% (Table 2, Figure 1). This corresponds to an NNT of 4.5 vaccinations to prevent 1 death. Similar results were observed for patients with infection onset ≥14 days after vaccination (65.1%; 95% CI: 27.2 to 83.2; AR reduction, 22.2%; NNT, 4.5) and across both vaccine subgroups (BNT162b2: 66%; 95% CI: 34.9 to 82.2; AR reduction, 21.2%; NNT, 4.7 and ChAdOx1: 78.4%; 95% CI: 30.4 to 93.3; AR reduction, 24.4%; NNT, 4.1; Supplementary Figures 1–3, Supplementary Table 5). Vaccinated participants had lower odds of hospital admission (OR, 0.80; 95% CI, .51 to 1.28; *P* = .36) and shorter length of stay (–1.89 days; 95% CI: –4.57 to .78; *P* = .17), but differences were not statistically significant, as predicted by power analysis (Table 2, Supplementary Tables 6 and 7). ChAdOx1 vaccinees demonstrated a slightly higher vaccine efficacy and a shorter hospital stay (Table 2). Median Ct values were 30.8 (IQR, 25.9–35.4) and 28.8 (IQR, 25.3–33.7; *P* = .053) for cases and controls, respectively (Table 1). The difference reached statistical significance in subgroup analysis for patients with infection onset ≥14 days after vaccination (30.6; IQR, 26.1–37.1 vs 28.2; IQR, 24.4–32.7; *P* = .005; Supplementary Table 2).

**Table 2.** Vaccine Effectiveness

Patient Group	Death	P Value	Admission	P Value	Length of stay	P Value
Entire cohort,^a^ N = 119	69.3 (45.8–82.6)	<.001	0.80 (0.51–1.28)	.36	–1.89 (–4.57 to 0.78)	.17
Vaccination ≥14 days,^b^ N = 62 (3)	65.1 (27.2–83.2)	.005	0.57 (0.30–1.09)	.09	–2.36 (–5.74 to 1.02)	.17
BNT162b2,^c^ N = 79	66.0 (34.9–82.2)	.001	0.75 (0.43–1.31)	.31	–0.92 (–3.83 to 1.98)	.53
ChAdOx1,^d^ N = 40	78.4 (30.4–93.3)	.01	0.80 (0.35–1.81)	.59	–3.98 (–9.45 to 1.58)	.15

Vaccine effectiveness estimates are reported as (1 – hazard ratio) × 100 (95% confidence interval [CI]) for death, odds ratio (95% CI) for admission to hospital, and difference in days (95% CI) for length of stay in hospital.

^a^Adjusted for ethnicity, chronic respiratory disease, diabetes, and chronic neurological disease.

^b^Adjusted for immunosuppression, chronic respiratory disease, renal dialysis, chronic neurological disease, active solid organ malignancy, and rheumatological disease.

^c^Adjusted for chronic neurological disease.

^d^Adjusted for chronic respiratory disease.

**Figure 2.**
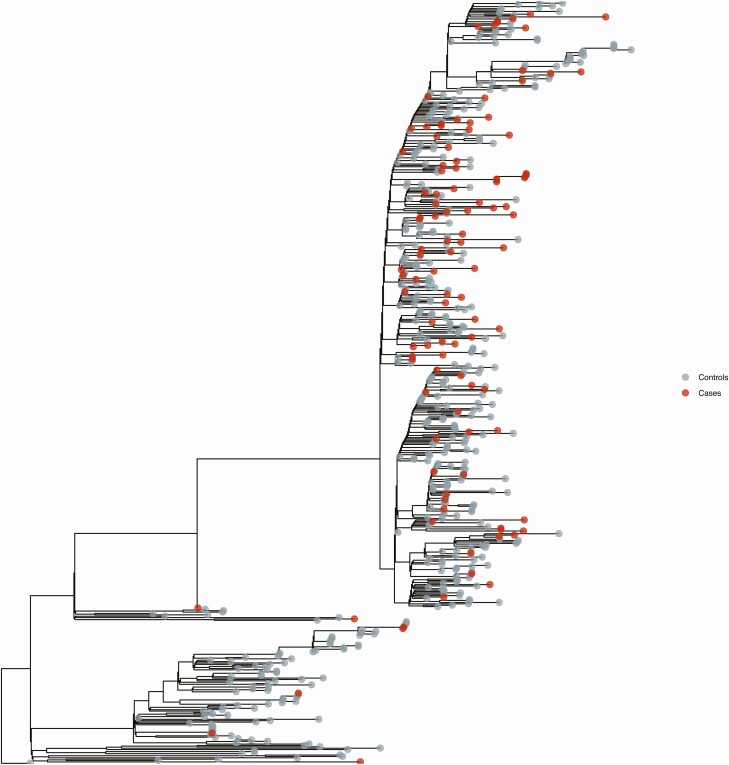
Maximum likelihood phylogeny of successfully sequenced samples (N = 554) using a generalized time-reversible model. The tree is routed on the reference strain MN908947.3, and each branch supported by 1000 bootstraps.

**Figure 3.**
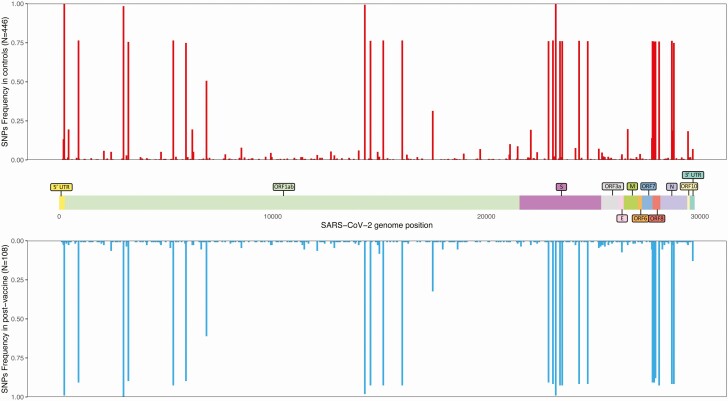
SNP frequency from successfully sequenced samples (N = 554) from cases and controls across the annotated SARS-CoV-2 MN908947.3 references genome. Abbreviations: SARS-CoV-2; severe acute respiratory syndrome coronavirus 2 SNP, single-nucleotide polymorphism.

Illumina and Nanopore sequencing were used for 527 of 595 (88.6%) and 68 of 595 (11.4%) samples, respectively. In total, 108 of 119 (90.8%) cases and 446 of 476 (93.7%) controls met quality criteria for further analysis (493 of 527, 93.5% for Illumina and 61 of 68, 89.7% for Nanopore). Median genome coverage was 97.7% (IQR, 94.1%–98.8%; range, 55%–99.6%). The maximum likelihood phylogeny of sequenced samples is shown in Figure 2. We found no clustering of viral sequences from post-vaccine cases, and no cases were associated with abnormal branch length. The frequency of common single-nucleotide polymorphisms (shared by more than 20% of individuals in either group) was no different between post-vaccine and controls (Figure 3). Only 1 potential vaccine-escape mutation, E484K, was found, and that was in an immunocompromised patient infected with the B.1.525 lineage 4 days after the first dose of BNT162b2 (Supplementary Table 8). The mean number of MVAs was not statistically different between the 2 groups (cases = 2.6, controls = 2.2, *P* = .17). GISAID accession numbers for all sequenced samples with ≥90% coverage are included in the Supplementary Materials.

## DISCUSSION

In this study, we describe a cohort of BNT162b2 or ChAdOx1 vaccinated multimorbid patients who developed COVID-19 predominantly from the B.1.1.7 lineage post–first vaccination. In this cohort, 1 life was saved every 4 to 5 vaccinations. Genomic analysis identified 1 patient with an escape mutation (E484K; B.1.525 lineage) who developed COVID-19 4 days post-vaccination.

We describe a real-world setting in which a mass vaccination campaign was rolled out during a pandemic. We therefore acknowledge that a proportion of cases would have been infected before vaccination and, therefore, were not strictly post-vaccination infections [25]. Yet, this is more of a theoretical concern, rather than a factor that confounds our results. Our data strongly suggest that these patients have a much more favorable outcome to their matched counterparts, whether they had “preventative” (pre-infection) or “therapeutic” (post-infection) vaccination.

Our data indirectly questions whether paucisymptomatic/asymptomatic patients should be denied vaccination, as we have demonstrated a survival benefit in patients who developed COVID-19 shortly after being vaccinated. Additional studies are required to explore this hypothesis. Figure 1 suggests that the mortality benefit from vaccination occurs immediately after COVID-19 infection, as Kaplan-Meier curves diverge early, suggesting rapid protection. Our data corroborate the latest Public Health England vaccine effectiveness report, suggesting reduced risk of death at the population level being measurable within the first 2-week period after vaccination, while reduced risk of hospitalization is only observed after the initial 2 weeks [7]. A nationwide study from Israel also demonstrated that reduction in deaths is achieved earlier than other outcomes [5]. A cluster study in US care homes showed that protection from a composite outcome of death or hospitalization was observed within the first week after vaccination [26]. Evidence is suggestive that a substantial mortality benefit is obtainable early after first vaccination, while avoidance of hospitalizations or cases might require additional time. Our study strongly supports the practice of delaying second vaccination to provide a greater degree of protection at a population level in settings where vaccines are limited.

We did not demonstrate a statistically significant reduction in hospitalizations and length of hospital stay after vaccination in contrast with previous studies [7, 8], as our cohort was clearly underpowered for these outcomes. Additionally, our population mainly consisted of multimorbid patients who presented to the emergency department with COVID-19 symptoms and therefore with a high baseline probability of admission compared with studies that evaluated patients at the community level. Our cohort also included patients within the first 14 days of vaccination, during which literature suggests that there is still minimal effect from vaccination [7]. This might have diluted our findings.

Our study is subject to multiple limitations. Our results only apply to the vaccines studied and to B.1.1.7 lineage. Our study population might not be representative of most institutions, given the high proportion of patients with rare conditions. Despite matching, we cannot completely eliminate residual confounding, especially with regard to the fact that our cases had been offered and accepted vaccination, while our controls might not have been offered or accepted vaccination. We elected to match for age and gender only to allow a bigger sample size. Some nonstatistically significant differences were observed. Additional matching would have put us at risk of overmatching, which can actually increase confounding [19].

We did not adjust for baseline COVID-19 serology or for previous history of infection, as this was not an interventional study, nor was the latter consistently documented. Natural infection is associated with a good degree of protection; therefore, we would not expect many of our patients to have had previous episodes [27]. Additionally, it is unclear whether previous infection would increase or decrease vaccination uptake; therefore, we cannot comment on the direction of potential confounding. Finally, a proportion of our cohort (between 30 September and 7 December) did not have the opportunity to be vaccinated and therefore develop post-vaccine infection. They were also less likely to be infected with the B.1.1.7 lineage. We decided to include them as controls due to power considerations and to aid the genomic analysis. Given that breakthroughs in COVID-19 therapeutics preceded our study period and that management of COVID-19 patients in the RFH did not substantially change throughout this time, we consider them an appropriate control for our study, with comparable background risk of mortality.

In summary, this study serves to inform clinicians and policy makers that vaccination with BNT162b2 or ChAdOx1 constitutes a powerful tool against the B.1.1.7 variant of concern. Although our results were reassuring for viral genomic changes post-vaccination, further surveillance of the impact of vaccine-escape mutations in vaccinees is required.

## Supplementary Data

Supplementary materials are available at *Clinical Infectious Diseases* online. Consisting of data provided by the authors to benefit the reader, the posted materials are not copyedited and are the sole responsibility of the authors, so questions or comments should be addressed to the corresponding author.

ciab714_suppl_Supplementary_Tables_Figures

ciab714_suppl_Supplementary_Appendix

## Notes

***Author contributions.*** I. B. conceived the study, determined study design, and performed the literature search. I. B. and F. A. T. B. wrote the study’s draft protocol and methodology, which was edited and approved by all authors. I. B., T. W. M., T. H., D. I.-T., and J. H. investigated, curated, and analyzed the clinical data. F. A. T. B. and J. B. investigated, curated, and analyzed the genomic data. R. W., S. R., J. A. G.-A., C. A. W., N. B., and M. C. sequenced the clinical samples. T. W. M. and J. B. supervised the study. J. B. acquired funding. I. B., T. W. M., F. A. T. B., and J. B. verified the underlying clinical and genomic data. I. B. wrote the original draft. All authors edited the manuscript for important intellectual content. All authors had full access to the deidentified and aggregated data in the study, approved the final version of the manuscript, and are responsible for the decision to submit for publication.

***Acknowledgments.*** We thank all study participants. We thank Darren Jones for assisting with extraction of the clinical data and Eirini Koutoumanou for assisting with statistical analysis.

***Disclaimer.*** The funders of this study had no role in study design; collection, analysis, and interpretation of data; writing of the manuscript; and the decision to submit the manuscript for publication.

***Financial support*.** This work was supported by funding awarded to J. B. for conducting the COG-UK Hospital-Onset COVID-19 Infections Wellcome Trust Study through grants from UKRI, the Wellcome Trust, and the John Black Charitable Foundation. This study was made possible through the COVID-19 Genomics UK (COG-UK) Consortium, supported by funding from the Medical Research Council part of UK Research and Innovation (UKRI), the National Institute of Health Research, and Genome Research Limited, operating as the Wellcome Sanger Institute.

**Declarations of interest**
