# Supplementary material for: Post-Vaccination Coronavirus Disease 2019: A Case-Control Study and Genomic Analysis of 119 Breakthrough Infections in Partially Vaccinated Individuals
Source: Clin Infect Dis. 2021 Aug 19;75(2):305–13. doi: 10.1093/cid/ciab714 (PMC8513403; doi:10.1093/cid/ciab714)
Supplement: ciab714_suppl_Supplementary_Tables_Figures [file ciab714_suppl_supplementary_tables_figures.docx]

| **Supplementary table 1: Temporal breakdown of post-vaccination cases** | |
| --- | --- |
| Days after first vaccination | Number of cases |
| 1 - 7 | 25 (21%) |
| 8 - 14 | 37 (31∙1%) |
| 15 - 21 | 21 (17∙6%) |
| 22 - 28 | 14 (11∙8%) |
| 29 - 35 | 9 (7∙6%) |
| >35 | 13 (10∙9%) |
| Total | 119 (100%) |
| Days after second vaccination | Number of cases |
| 1 - 7 | 0 (0%) |
| 8 - 14 | 1 (33∙3%) |
| 15 - 21 | 0 (0%) |
| 22 - 28 | 1 (33∙3%) |
| 29 - 35 | 0 (0%) |
| >35 | 1 (33∙3%) |
| Total | 3 (100%) |

Supplementary table 1: Temporal breakdown of post-vaccination infections.

| **Supplementary table 2: Subgroup analysis – COVID-19 ≥14 days after vaccination** | | | |
| --- | --- | --- | --- |
| Variables | Cases (N = 62) | Controls (N = 248) | P value |
| Age | 82 (66∙75 – 87) | 80 (68 – 87) | 0.77 |
| <30 | 0 (0%) | 0 (0%) | 1 |
| 30 - 39 | 2 (3∙2%) | 8 (3∙2%) |  |
| 40 - 49 | 1 (1∙6%) | 3 (1∙2%) |  |
| 50 - 59 | 8 (12∙9%) | 34 (13∙7%) |  |
| 60 - 69 | 5 (8∙1%) | 21 (8∙5%) |  |
| 70 - 79 | 11 (17∙7%) | 51 (20∙6%) |  |
| >80 | 35 (56∙5%) | 131 (52∙8%) |  |
| Sex |  |  |  |
| Male | 38 (61∙3%) | 152 (61∙3%) | 1 |
| Female | 24 (38∙7%) | 96 (38∙7%) |  |
| Ethnicity |  |  |  |
| White | 47 (75∙8%) | 166 (66∙9%) | 0.30 |
| Asian | 12 (19∙4%) | 52 (21%) |  |
| Black | 3 (4∙8%) | 22 (8∙9%) |  |
| Mixed/Other | 0 (0%) | 8 (3∙2%) |  |
| MID Quartile^a^ |  |  |  |
| 1^st^ | 9 (14∙5%) | 53 (21∙4%) | 0.65 |
| 2^nd^ | 14 (22∙6%) | 56 (22∙6%) |  |
| 3^rd^ | 19 (30∙6%) | 64 (25∙8%) |  |
| 4^th^ | 20 (32∙3%) | 75 (30∙2%) |  |
| Nursing/Care home resident | 3 (4∙8%) | 13 (5∙2%) | 1 |
| Variant^b^ |  |  |  |
| B.1.1.7 | 52 (83∙9%) | 188 (75∙8%%) | 0.001 |
| Other | 1 (1∙6%) | 45 (18∙1%) |  |
| Low quality | 9 (14∙5%) | 15 (6∙1%) |  |
| Days from vaccination^c^ | 24 (17 – 34) | N/A |  |
| Pregnancy | 0 (0%) | 1 (0∙4%) | 1 |
| Chronic renal disease | 7 (11∙3%) | 39 (15∙7%) | 0.43 |
| Immunosuppression | 13 (21%) | 26 (10∙5%) | 0.03 |
| Obesity | 2 (3∙2%) | 13 (5∙2%) | 0.74 |
| Transplant | 3 (4∙8%) | 6 (2∙4%) | 0.39 |
| Asplenia | 0 (0%) | 1 (0∙4%) | 1 |
| HIV | 0 (0%) | 0% | N/A |
| Chronic respiratory disease | 7 (11∙3%) | 46 (18∙5%) | 0.19 |
| Asthma | 4 (6∙5%) | 26 (10∙5%) | 0.47 |
| Chronic cardiac disease | 30 (48∙4%) | 135 (54∙4%) | 0.40 |
| Renal dialysis | 2 (3∙2%) | 23 (9∙3%) | 0.19 |
| Chronic liver disease | 6 (9∙7%) | 25 (10∙1%) | 1 |
| Diabetes | 21 (33∙9%) | 91 (36∙7%) | 0.77 |
| Chronic neurological disease | 8 (12∙9%) | 57 (23%) | 0.12 |
| Active solid organ malignancy | 11 (17∙7%) | 28 (11∙3%) | 0.2 |
| Haemotological disease | 5 (8∙1%) | 12 (4∙8%) | 0.35 |
| Rheumatological disease | 10 (16∙1%) | 20 (8∙1%) | 0.09 |
| Dementia | 9 (14∙5%) | 43 (17∙3%) | 0.71 |
| Ct value^d^ | 30∙6 (26∙1 – 37∙1) | 28∙2 (24∙4 – 32∙7) | 0.005 |
| Admission to hospital | 42 (67∙7%) | 193 (77∙8%) | 0.10 |
| Length of stay in hospital^e^ | 5∙5 (3 – 13) | 8 (5 – 15) | 0.18 |
| Death | 8 (12∙9%) | 87 (35∙1%) | 0.001 |
| Continuous variables are presented as median (IQR), categorical variables as N (%).^a^The first quartile represents the least deprived participants. ^b^Other includes wild-type COVID-19 or variants that have not been characterized as variants of concern Not all samples met sequencing quality criteria. ^c^Indicates days since first vaccination. ^d^N = 57 for cases and N = 211 for controls, excludes samples tested in the Aptima platform ^e^N = 38 for cases and N = 130 for controls, only includes patient that were admitted and survived their admission. | | | |

Supplementary table 2: Summary characteristics of cases and controls in the subgroup of patients with COVID-19 infection with onset ≥14 days after vaccination.

| **Supplementary table 3: Subgroup analysis – COVID-19 after BNT162b2** | | | |
| --- | --- | --- | --- |
| Variables | Cases (N = 79) | Controls (N = 316) | P value |
| Age | 82 (65 – 86) | 80 (66∙25 – 87) | 0.73 |
| <30 | 2 (2∙5%) | 9 (2∙8%) | 1 |
| 30 - 39 | 2 (2∙5%) | 7 (2∙2%) |  |
| 40 - 49 | 2 (2∙5%) | 9 (2∙8%) |  |
| 50 - 59 | 9 (11∙5%) | 36 (11∙4%) |  |
| 60 - 69 | 5 (6∙3%) | 21 (6∙6%) |  |
| 70 - 79 | 14 (17∙7%) | 67 (21∙2%) |  |
| >80 | 45 (57%) | 167 (52∙8%) |  |
| Sex |  |  |  |
| Male | 48 (60∙8%) | 192 (60∙8%) | 1 |
| Female | 31 (39∙2%) | 124 (39∙2%) |  |
| Ethnicity |  |  |  |
| White | 58 (73∙4%) | 225 (71∙2%) | 0.55 |
| Asian | 16 (20∙3%) | 61 (19∙3%) |  |
| Black | 5 (6∙3%) | 22 (7%) |  |
| Mixed/Other | 0 (0%) | 8 (2∙5%) |  |
| MID Quartile^a^ |  |  |  |
| 1^st^ | 11 (13∙9%) | 63 (19∙9%) | 0.46 |
| 2^nd^ | 16 (20∙3%) | 62 (19∙6%) |  |
| 3^rd^ | 28 (35∙4%) | 88 (27∙9%) |  |
| 4^th^ | 24 (30∙4%) | 103 (32∙6%) |  |
| Nursing/Care home resident | 5 (6∙3%) | 14 (4∙4%) | 0.56 |
| Variant^b^ |  |  |  |
| B.1.1.7 | 69 (87∙3%) | 224 (70∙8%) | <0.001 |
| Other | 4 (5∙1%) | 70 (22∙2%) |  |
| Low quality | 6 (7∙6%) | 22 (7%) |  |
| Days from vaccination^c^ | 15 (9 -27) | N/A |  |
| Pregnancy | 0 (0%) | 8 (2∙5%) | 0.34 |
| Chronic renal disease | 11 (13∙9%) | 43 (13∙6%) | 1 |
| Immunosuppression | 12 (15∙2%) | 37 (11∙7%) | 0.45 |
| Obesity | 3 (3∙8%) | 18 (5∙7%) | 0.78 |
| Transplant | 2 (2∙5%) | 6 (1∙9%) | 0.66 |
| Asplenia | 0 (0%) | 2 (0∙6%) | 1 |
| HIV | 0 (0%) | 0 (0%) | N/A |
| Chronic respiratory disease | 11 (13∙9%) | 60 (19%) | 0.33 |
| Asthma | 6 (7∙6%) | 29 (9∙2%) | 0.83 |
| Chronic cardiac disease | 40 (50∙6%) | 178 (56∙3%) | 0.38 |
| Renal dialysis | 4 (5∙1%) | 19 (6%) | 1 |
| Chronic liver disease | 6 (7∙6%) | 26 (8∙2%) | 1 |
| Diabetes | 22 (27∙8%) | 110 (34∙8%) | 0.29 |
| Chronic neurological disease | 11 (13∙9%) | 73 (23∙1%) | 0.09 |
| Active solid organ malignancy | 11 (13∙9%) | 33 (10∙4%) | 0.42 |
| Haemotological disease | 6 (7∙6%) | 16 (5∙1%) | 0.41 |
| Rheumatological disease | 10 (12∙7%) | 28 (8∙9%) | 0.29 |
| Dementia | 12 (15∙2%) | 55 (17∙4%) | 0.74 |
| Ct value^d^ | 30∙7 (26∙1 – 36∙3) | 28∙6 (25∙3 – 34∙0) | 0.05 |
| Admission to hospital | 56 (70∙9%) | 244 (77∙2%) | 0.24 |
| Length of stay in hospital^e^ | 7 (4 -11∙5) | 8 (4 – 15) | 0.5 |
| Death | 10 (12∙7%) | 107 (33∙9%) | <0.001 |
| Continuous variables are presented as median (IQR), categorical variables as N (%).^a^The first quartile represents the least deprived participants. ^b^Other includes wild-type COVID-19 or variants that have not been characterized as variants of concern. Not all samples met sequencing quality criteria ^c^Indicates days since first vaccination. ^d^N = 73 for cases and N = 261 for controls, excludes samples tested in the Aptima platform. ^e^N = 49 for cases and N = 164 for controls, only includes patient that were admitted and survived their admission. | | | |

Supplementary table 3: Summary characteristics of cases and controls in the subgroup of patients with COVID-19 infection after the BNT162b2 mRNA vaccine.

| **Supplementary table 4: Subgroup analysis – COVID-19 after ChAdOx1** | | | |
| --- | --- | --- | --- |
| Variables | Post Astrazeneca (N = 40) | Matched control group 4:1 (N = 160) | P value |
| Age | 74∙5 (65∙25 – 84∙75) | 75 (66 – 83) | 0.81 |
| <30 | 0 (0%) | 0 (0%) | 1 |
| 30 - 39 | 1 (2∙5%) | 4 (2∙5%) |  |
| 40 - 49 | 0 (0%) | 1 (0∙6%) |  |
| 50 - 59 | 6 (15%) | 23 (14∙4%) |  |
| 60 - 69 | 6 (15%) | 26 (16∙3%) |  |
| 70 - 79 | 13 (32∙5%) | 49 (30∙6%) |  |
| >80 | 14 (35%) | 57 (35∙6%) |  |
| Sex |  |  |  |
| Male | 20 (50%) | 80 (50%) | 1 |
| Female | 20 (50%) | 80 (50%) |  |
| Ethnicity |  |  |  |
| White | 30 (75%) | 98 (61∙2%) | 0.24 |
| Asian | 9 (22∙5%) | 43 (26∙9%) |  |
| Black | 1 (2∙5%) | 11 (6∙9%) |  |
| Mixed/Other | 0 (0%) | 8 (5%) |  |
| MID Quartile^a^ |  |  |  |
| 1^st^ | 9 (22∙5%) | 43 (26∙9%) | 0.89 |
| 2^nd^ | 13 (32∙5%) | 43 (26∙9%) |  |
| 3^rd^ | 9 (22∙5%) | 39 (24∙3%) |  |
| 4^th^ | 9 (22∙5%) | 35 (21∙9%) |  |
| Nursing/Care home resident | 1 (2∙5%) | 8 (5%) | 0.69 |
| Variant^b^ |  |  |  |
| B.1.1.7 | 31 (77∙5%) | 117 (73∙1%) | 0.17 |
| Other | 4 (10%) | 35 (21∙9%) |  |
| Low quality | 5 (12∙5%) | 8 (5%) |  |
| Days from vaccination^c^ | 11 (5∙5 – 20∙75) | N/A |  |
| Pregnancy | 0 (0%) | 1 (0∙6%) | 1 |
| Chronic renal disease | 5 (12∙5%) | 19 (11∙9%) | 1 |
| Immunosuppression | 4 (10%) | 22 (13∙8%) | 0.79 |
| Obesity | 2 (5%) | 13 (8∙1%) | 0.74 |
| Transplant | 2 (5%) | 5 (3∙1%) | 0.63 |
| Asplenia | 0 (0%) | 0 (0%) | N/A |
| HIV | 0 (0%) | 1 (0∙6%) | 1 |
| Chronic respiratory disease | 3 (7∙5%) | 30 (18∙8%) | 0.1 |
| Asthma | 2 (5%) | 16 (10%) | 0.54 |
| Chronic cardiac disease | 19 (47∙5%) | 79 (49∙4%) | 0.86 |
| Renal dialysis | 4 (10%) | 12 (7∙5%) | 0.53 |
| Chronic liver disease | 2 (5%) | 12 (7∙5%) | 0.74 |
| Diabetes | 13 (32∙5%) | 61 (38∙1%) | 0.59 |
| Chronic neurological disease | 8 (20%) | 37 (23∙1%) | 0.83 |
| Active solid organ malignancy | 4 (10%) | 17 (10∙6%) | 1 |
| Haemotological disease | 3 (7∙5%) | 13 (8∙1%) | 1 |
| Rheumatological disease | 3 (7∙5%) | 12 (7∙5%) | 1 |
| Dementia | 6 (15%) | 28 (17∙5%) | 0.82 |
| Ct value^d^ | 30∙9 (24∙6 – 34∙4) | 29∙2 (25∙3 – 33∙3) | 0.56 |
| Length of stay in hospital^e^ | 6 (3 – 12) | 11 (5 – 18) | 0.04 |
| Admission to hospital | 30 (75%) | 127 (79∙4%) | 0.53 |
| Death | 3 (7∙5%) | 51 (31∙9%) | 0.001 |
| Continuous variables are presented as median (IQR), categorical variables as N (%).^a^The first quartile represents the least deprived participants. ^b^Other includes wild-type COVID-19 or variants that have not been characterized as variants of concern. Not all samples met sequencing quality criteria. ^c^Indicates days since first vaccination. ^d^N = 39 for cases and N = 138 for controls, excludes samples tested in the Aptima platform. ^e^N = 29 for cases and N = 95 for controls, only includes patient that were admitted and survived their admission. | | | |

Supplementary table 4: Summary characteristics of cases and controls in the subgroup of patients with COVID-19 infection after the ChAdOx1 nCoV-19 vaccine.

**Supplementary Figure 1**


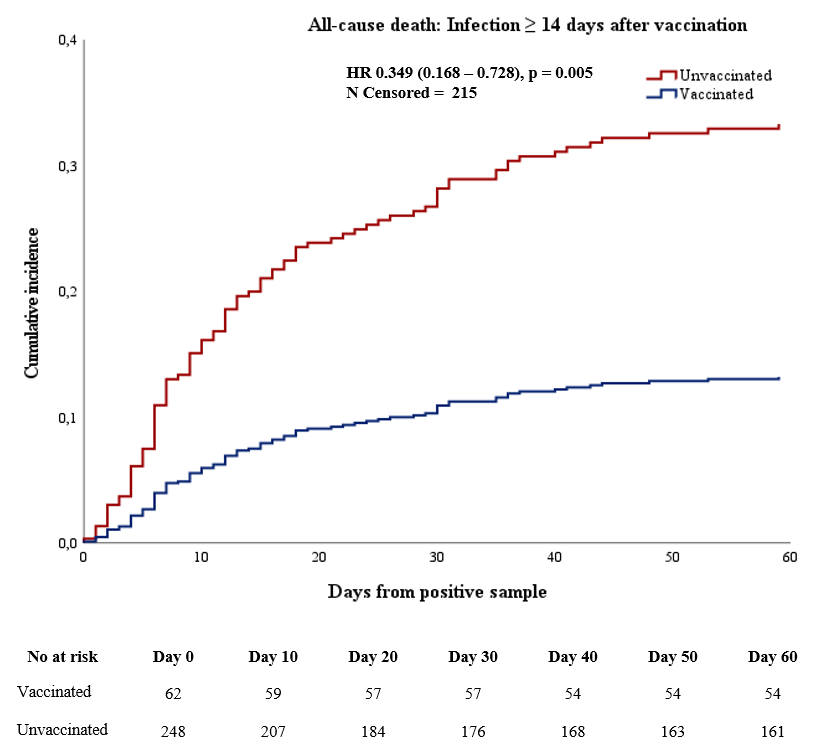


Supplementary figure 1: Cumulative incidence curves (1 minus hazard ratio) for all-cause death before 60 days for the subgroup of patients with infection onset ≥14 days after vaccination, starting from the day of the index positive COVID-19 sample. Numbers at risk at each time point and numbers censored are also shown.

**Supplementary Figure 2**


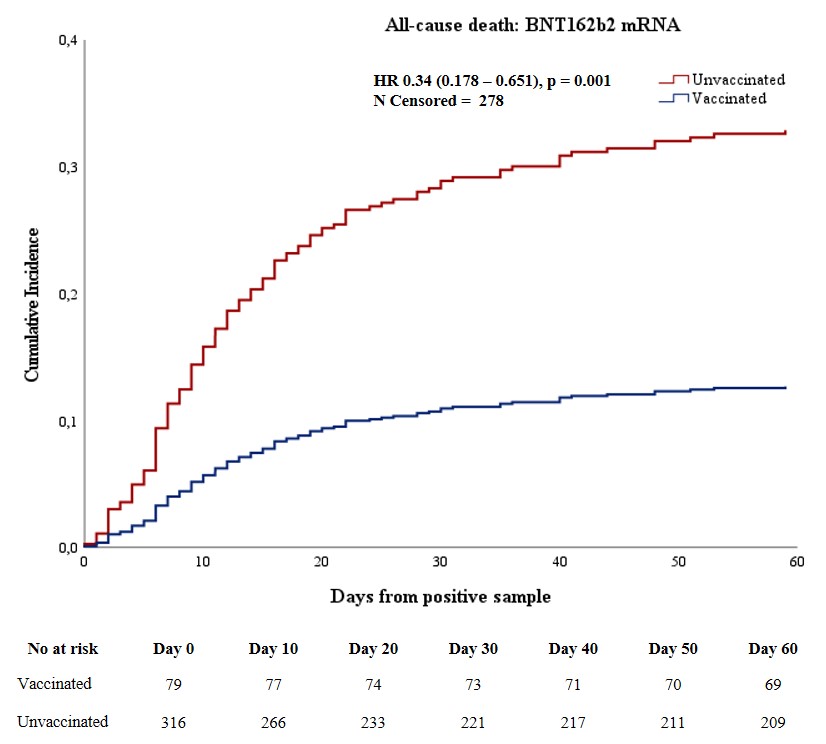


Supplementary Figure 2: Cumulative incidence curves (1 minus hazard ratio) for all-cause death before 60 days for the subgroup of patients with previous BNT162b2 vaccination, starting from the day of the index positive COVID-19 sample. Numbers at risk at each time point and numbers censored are also shown.

**Supplementary Figure 3:**


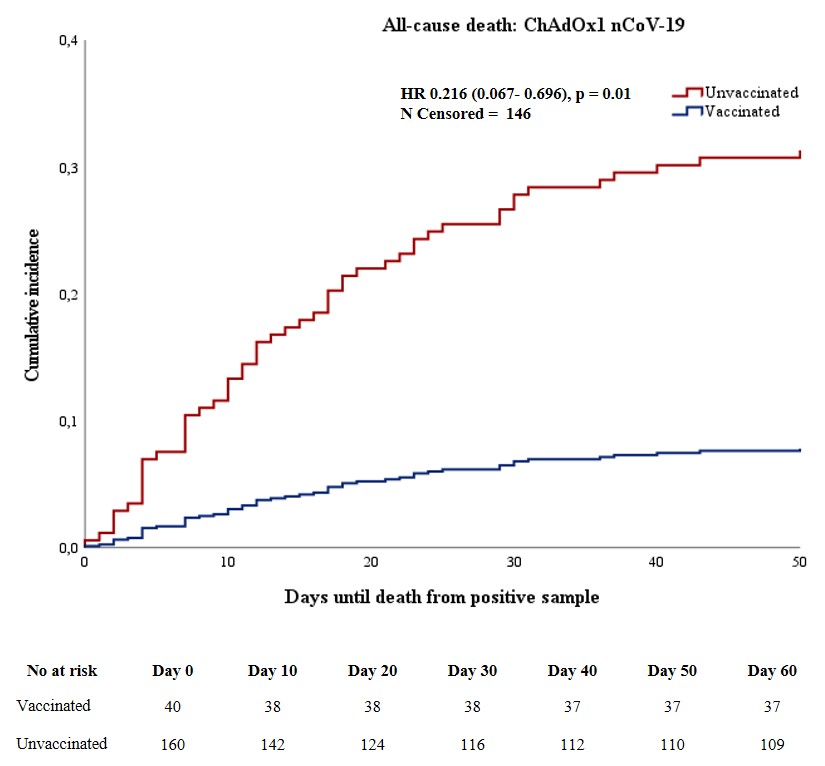


Supplementary figure 3: Cumulative incidence curves (1 minus hazard ratio) for all-cause death before 60 days for the subgroup of patients with previous ChAdOx1 vaccination, starting from the day of the index positive COVID-19 sample. Numbers at risk at each time point and numbers censored are also shown.

| **Supplementary table 5: Cox regression results for death** | | | | | | | | |
| --- | --- | --- | --- | --- | --- | --- | --- | --- |
|  | All  (N= 595) | p | Infection ≥14 days after vaccination  (N = 310) | p | BNT162b2  (N = 395) | p | ChAdOx1  (N = 200) | p |
| Previous vaccination | 0∙31 (0∙17–0∙54) | <0.001 | 0∙35 (0∙17-0∙73) | 0.005 | 0∙34 (0∙18 – 0∙65) | 0.001 | 0∙22 (0∙07-0∙70) | 0.01 |
| Ethnicity |  |  |  |  |  |  |  |  |
| White | Baseline group |  | N/I |  | N/I |  | N/I |  |
| Asian | 0∙65 (0∙43-0∙98) | 0.04 | N/I |  | N/I |  | N/I |  |
| Black | 0∙72 (0∙37-1∙37) | 0.31 | N/I |  | N/I |  | N/I |  |
| Mixed/Other | 1∙20 (0∙56-2∙57) | 0.65 | N/I |  | N/I |  | N/I |  |
| Immunosuppression | N/I |  | 1∙37 (0∙73-2∙56) | 0.33 | N/I |  | N/I |  |
| Chronic respiratory disease | 1∙32 (0∙92-1∙88) |  | 1∙26 (0∙77-2∙07) | 0.36 | N/I |  | 1∙50 (0∙80-2∙80) | 0.20 |
| Renal dialysis | N/I |  | 1∙49 (0∙76-2∙93) | 0.25 | N/I |  | N/I |  |
| Diabetes | 1∙49 (1∙09-2∙04) | 0.01 | N/I |  | N/I |  | N/I |  |
| Chronic neurological disease | 1∙86 (1∙35-2∙56) | <0.001 | 2∙16 (1∙41-3∙30) | <0.001 | 2∙16 (1∙47-3∙27) | <0.001 | N/I |  |
| Active solid organ malignancy | N/I |  | 1∙37 (0∙77-2∙44) | 0.28 | N/I |  | N/I |  |
| Chronic Rheumatological disease | N/I |  | 0∙70 (0∙30-1∙59) | 0.39 | N/I |  | N/I |  |
| Numbers indicate Hazard ratios and 95%CIs. N/I: not included | | | | | | | |  |

Supplementary Table 5: Multivariable Cox regression results for death for the entire cohort and prespecified subgroups. Variables included if p <0∙2 in univariable comparison (Table 1, Supplementary tables 2-4).

| **Supplementary table 6: Logistic regression results for admission to hospital** | | | | | | | | |
| --- | --- | --- | --- | --- | --- | --- | --- | --- |
|  | All  (N= 595) | p | Infection ≥14 days after vaccination  (N = 310) | p | BNT162b2  (N = 395) | p | ChAdOx1  (N = 200) | p |
| Previous vaccination | 0∙80 (0∙51-1∙28) | 0.36 | 0∙57 (0∙30-1∙09) | 0.09 | 0∙75 (0∙43-1∙31) | 0.31 | 0∙80 (0∙35 - 1∙81) | 0.59 |
| Ethnicity |  |  |  |  |  |  |  |  |
| White | Baseline group |  | N/I |  | N/I |  | N/I |  |
| Asian | 0∙74 (0∙46-1∙18) | 0.74 | N/I |  | N/I |  | N/I |  |
| Black | 0∙56 (0∙27-1∙17) | 0.12 | N/I |  | N/I |  | N/I |  |
| Mixed/Other | 1∙74 (0∙38- 7∙92) | 0.47 | N/I |  | N/I |  | N/I |  |
| Immunosuppression | N/I |  | 2∙70 (0∙99-7∙29) | 0.05 | N/I |  | N/I |  |
| Chronic respiratory disease | 1∙84 (1∙02-3∙32) | 0.04 | 1∙85 (0∙81-4∙25) | 0.15 | N/I |  | 1∙24 (0∙47-3∙26) | 0.66 |
| Renal dialysis | N/I |  | 0∙40 (0∙16 – 0∙98) | 0.05 | N/I |  | N/I |  |
| Diabetes | 1∙51 (0∙98-2∙33) | 0.06 | N/I |  | N/I |  | N/I |  |
| Chronic neurological disease | 1∙63 (0∙97-2∙75) | 0.07 | 1∙18 (0∙60-2∙33) | 0.63 | 1∙72 (0∙92-3∙22) | 0.09 | N/I |  |
| Active solid organ malignancy | N/I |  | 1∙06 (0∙45-2∙52) | 0.90 | N/I |  | N/I |  |
| Chronic Rheumatological disease | N/I |  | 0∙37 (0∙15-0∙92) | 0.03 | N/I |  | N/I |  |
| Numbers indicate odds ratios and 95%CIs. N/I: not included | | | | | | | | |

Supplementary table 6: Multivariable logistic regression results for admission to hospital for the entire cohort and prespecified subgroups. Variables included if p <0∙2 in univariable comparison (Table 1, Supplementary tables 2-4).

| **Supplementary table 7: Liner regression results for length of stay in hospital** | | | | | | | | | |
| --- | --- | --- | --- | --- | --- | --- | --- | --- | --- |
|  | All  (N= 595) | | p | Infection ≥14 days after vaccination  (N = 310) | p | BNT162b2  (N = 395) | p | ChAdOx1  (N = 200) | p |
| Previous vaccination | | -1∙89 (-4∙57-0∙78) | 0.17 | -2∙36 (-5∙74-1∙02) | 0.17 | -0∙92 (-3∙83-1∙98) | 0.53 | -3∙98 (-9∙45-1∙58) | 0.15 |
| Ethnicity | |  |  |  |  |  |  |  |  |
| White | | Baseline group |  | N/I |  | N/I |  | N/I |  |
| Asian | | -2∙78 (-4∙74-0∙58) | 0.13 | N/I |  | N/I |  | N/I |  |
| Black | | -2∙14 (-6∙53-7∙64) | 0.34 | N/I |  | N/I |  | N/I |  |
| Mixed/Other | | 1∙01 (-5∙63-7∙64) | 0.77 | N/I |  | N/I |  | N/I |  |
| Immunosuppression | | N/I |  | 1∙47 (-2∙99-5∙92) |  | N/I |  | N/I |  |
| Chronic respiratory disease | | 3∙71 (0∙89-6∙53) | 0.01 | 2∙35 (-1∙19-5∙89) | 0.19 | N/I |  | 7∙42 (1∙53-13∙31) | 0.01 |
| Renal dialysis | | N/I |  | -4∙73 (-9∙55-0∙19) | 0.06 | N/I |  | N/I |  |
| Diabetes | | 1∙77 (-0∙53-4∙06) | 0.13 | N/I |  | N/I |  | N/I |  |
| Chronic neurological disease | | 1∙11 (-1∙48-3∙71) | 0.40 | 1∙28 (-1∙98-4∙53) | 0.44 | 1∙63 (-1∙21-4∙47) | 0.26 | N/I |  |
| Active solid organ malignancy | | N/I |  | 2∙90 (-1∙22-7∙03) | 0.17 | N/I |  | N/I |  |
| Chronic Rheumatological disease | | N/I |  | -3∙24 (-8∙11-1∙63) | 0.19 | N/I |  | N/I |  |
| Numbers indicate difference in days and 95%CIs. N/I: not included | | | | | | | | | |

Supplementary table 7: Multivariable linear regression results length of stay in hospital for the entire cohort and prespecified subgroups. Variables included if p <0∙2 in univariable comparison (Table 1, Supplementary tables 2-4).

| **Supplementary Table 8: Incidence of clinically relevant mutations in the S gene** | | | |
| --- | --- | --- | --- |
| Mutation | Cases (N = 108) | Controls (N = 446) | p |
| del69.70 | 102 (94∙4%) | 341 (76∙5%) | <0.001 |
| del144 | 103 (95∙4%) | 340 (76∙2%) | <0.001 |
| del242.244 | 0 (0%) | 1 (0∙2%) | 1 |
| D80A | 0 (0%) | 0 (0%) | 1 |
| T95I | 0 (0%) | 1 (0∙2%) | 1 |
| D138Y | 0 (0%) | 0 (0%) | 1 |
| R190T | 0 (0%) | 0 (0%) | 1 |
| D215G | 0 (0%) | 0 (0%) | 1 |
| F220I | 0 (0%) | 0 (0%) | 1 |
| R237K | 0 (0%) | 0 (0%) | 1 |
| R246T | 0 (0%) | 0 (0%) | 1 |
| D253G | 0 (0%) | 1 (0∙2%) | 1 |
| K417T | 0 (0%) | 0 (0%) | 1 |
| L452R | 0 (0%) | 0 (0%) | 1 |
| S477N | 0 (0%) | 2 (0∙4%) | 1 |
| E484K | 1 (0∙9%) | 0 (0%) | 1 |
| N501Y | 100 (92∙6%) | 337 (75∙6%) | <0.001 |
| A570D | 100 (92∙6%) | 340 (76∙2%) | <0.001 |
| D614G | 107 (99∙1%) | 446 (100%) | 1 |
| H655Y | 0 (0%) | 0 (0%) | 1 |
| P681H | 101 (93∙5%) | 338 (75∙8%) | <0.001 |
| A701V | 0 (0%) | 2 (0∙4%) | 1 |
| D796H | 0 (0%) | 0 (0%) | 1 |
| 108/119 (90∙8%) and 446/476 (93∙7%) of total samples of cases and controls respectively met quality criteria for further analysis (493/527, 93.5% Illumina sequencing and 61/68, 89.7% Oxford Nanopore Technology).. Inclusion criteria 10x depth, >90% coverage. | | | |

Supplementary table 8: Incidence of clinically relevant mutations in the S gene.
