# Supplementary material for: Post-Vaccination Coronavirus Disease 2019: A Case-Control Study and Genomic Analysis of 119 Breakthrough Infections in Partially Vaccinated Individuals
Source: Clin Infect Dis. 2021 Aug 19;75(2):305–13. doi: 10.1093/cid/ciab714 (PMC8513403; doi:10.1093/cid/ciab714)
Supplement: ciab714_suppl_Supplementary_Appendix [file ciab714_suppl_supplementary_appendix.docx]

| GISAID Accession Numbers for samples with ≥90% coverage (N = 471) | |
| --- | --- |
| Primary Accession Number | Secondary Accession Number |
| EPI_ISL_1050469  EPI_ISL_1050471  EPI_ISL_1050472  EPI_ISL_1050473  EPI_ISL_1050475  EPI_ISL_1050478  EPI_ISL_1050479  EPI_ISL_1050482  EPI_ISL_1050486  EPI_ISL_1050487  EPI_ISL_1050491  EPI_ISL_1050493  EPI_ISL_1050494  EPI_ISL_1050495  EPI_ISL_1050496  EPI_ISL_1050499  EPI_ISL_1050500  EPI_ISL_1050503  EPI_ISL_1050504  EPI_ISL_1050506  EPI_ISL_1050507  EPI_ISL_1050508  EPI_ISL_1050512  EPI_ISL_1050519  EPI_ISL_1050521  EPI_ISL_1050525  EPI_ISL_1104662  EPI_ISL_1104665  EPI_ISL_1104669  EPI_ISL_1104670  EPI_ISL_1104671  EPI_ISL_1104680  EPI_ISL_1104682  EPI_ISL_1104684  EPI_ISL_1104685  EPI_ISL_1104691  EPI_ISL_1104694  EPI_ISL_1104695  EPI_ISL_1104696  EPI_ISL_1104701  EPI_ISL_1104702  EPI_ISL_1104703  EPI_ISL_1104704  EPI_ISL_1178009  EPI_ISL_1178011  EPI_ISL_1178017  EPI_ISL_1178018  EPI_ISL_1178019  EPI_ISL_1178020  EPI_ISL_1178021  EPI_ISL_1248000  EPI_ISL_1248008  EPI_ISL_1248009  EPI_ISL_1248013  EPI_ISL_1248016  EPI_ISL_1248019  EPI_ISL_1248021  EPI_ISL_1248026  EPI_ISL_1248030  EPI_ISL_1248036  EPI_ISL_1248042  EPI_ISL_1248045  EPI_ISL_1248054  EPI_ISL_1248061  EPI_ISL_1248062  EPI_ISL_1248064  EPI_ISL_1248066  EPI_ISL_1248070  EPI_ISL_1248078  EPI_ISL_1248079  EPI_ISL_1248099  EPI_ISL_1248100  EPI_ISL_1248107  EPI_ISL_1248109  EPI_ISL_1248110  EPI_ISL_1248111  EPI_ISL_1248122  EPI_ISL_1248135  EPI_ISL_1248136  EPI_ISL_1248149  EPI_ISL_1248153  EPI_ISL_1248158  EPI_ISL_1248162  EPI_ISL_1248164  EPI_ISL_1248165  EPI_ISL_1248166  EPI_ISL_1248167  EPI_ISL_1248170  EPI_ISL_1248178  EPI_ISL_1248184  EPI_ISL_1248189  EPI_ISL_1248195  EPI_ISL_1248197  EPI_ISL_1248198  EPI_ISL_1248212  EPI_ISL_1248218  EPI_ISL_1248220  EPI_ISL_1248225  EPI_ISL_1248228  EPI_ISL_1248230  EPI_ISL_1248232  EPI_ISL_1248236  EPI_ISL_1248244  EPI_ISL_1248264  EPI_ISL_1248269  EPI_ISL_1248284  EPI_ISL_1248287  EPI_ISL_1248289  EPI_ISL_1248292  EPI_ISL_1248300  EPI_ISL_1308974  EPI_ISL_1308979  EPI_ISL_1308989  EPI_ISL_1308995  EPI_ISL_1309001  EPI_ISL_1309002  EPI_ISL_1309003  EPI_ISL_1309005  EPI_ISL_1309011  EPI_ISL_1386831  EPI_ISL_665711  EPI_ISL_665720  EPI_ISL_679577  EPI_ISL_679580  EPI_ISL_679582  EPI_ISL_679586  EPI_ISL_679588  EPI_ISL_679593  EPI_ISL_679597  EPI_ISL_679603  EPI_ISL_679604  EPI_ISL_679606  EPI_ISL_679609  EPI_ISL_679610  EPI_ISL_679613  EPI_ISL_679614  EPI_ISL_679615  EPI_ISL_679617  EPI_ISL_679618  EPI_ISL_679619  EPI_ISL_704963  EPI_ISL_705007  EPI_ISL_705132  EPI_ISL_705232  EPI_ISL_705392  EPI_ISL_705396  EPI_ISL_724604  EPI_ISL_724605  EPI_ISL_724607  EPI_ISL_763381  EPI_ISL_763399  EPI_ISL_763442  EPI_ISL_763485  EPI_ISL_763578  EPI_ISL_763628  EPI_ISL_763700  EPI_ISL_763728  EPI_ISL_763730  EPI_ISL_763785  EPI_ISL_764037  EPI_ISL_764042  EPI_ISL_764045  EPI_ISL_764046  EPI_ISL_764048  EPI_ISL_764050  EPI_ISL_764055  EPI_ISL_764175  EPI_ISL_764384  EPI_ISL_764386  EPI_ISL_813908  EPI_ISL_813910  EPI_ISL_813914  EPI_ISL_813915  EPI_ISL_813920  EPI_ISL_813921  EPI_ISL_813922  EPI_ISL_813934  EPI_ISL_813935  EPI_ISL_813943  EPI_ISL_813944  EPI_ISL_813945  EPI_ISL_813946  EPI_ISL_813947  EPI_ISL_813949  EPI_ISL_813950  EPI_ISL_813952  EPI_ISL_813956  EPI_ISL_813957  EPI_ISL_813958  EPI_ISL_813959  EPI_ISL_813969  EPI_ISL_819874  EPI_ISL_819889  EPI_ISL_819893  EPI_ISL_819919  EPI_ISL_819924  EPI_ISL_819925  EPI_ISL_819926  EPI_ISL_819927  EPI_ISL_819929  EPI_ISL_819937  EPI_ISL_819943  EPI_ISL_819948  EPI_ISL_819958  EPI_ISL_819962  EPI_ISL_819964  EPI_ISL_819965  EPI_ISL_819981  EPI_ISL_819983  EPI_ISL_819989  EPI_ISL_819995  EPI_ISL_819998  EPI_ISL_820000  EPI_ISL_820009  EPI_ISL_820014  EPI_ISL_820018  EPI_ISL_820033  EPI_ISL_820037  EPI_ISL_820048  EPI_ISL_820054  EPI_ISL_820061  EPI_ISL_820063  EPI_ISL_820066  EPI_ISL_820081  EPI_ISL_820083  EPI_ISL_820086  EPI_ISL_820099  EPI_ISL_820111  EPI_ISL_820113  EPI_ISL_820115  EPI_ISL_820118  EPI_ISL_820122  EPI_ISL_820123  EPI_ISL_820125  EPI_ISL_820127  EPI_ISL_820128  EPI_ISL_820204  EPI_ISL_820209  EPI_ISL_820216  EPI_ISL_820226  EPI_ISL_820251  EPI_ISL_820259  EPI_ISL_820309  EPI_ISL_820318  EPI_ISL_820326  EPI_ISL_820333  EPI_ISL_820337  EPI_ISL_820345  EPI_ISL_820376  EPI_ISL_820383  EPI_ISL_820396  EPI_ISL_839040  EPI_ISL_839042  EPI_ISL_839043  EPI_ISL_839044  EPI_ISL_839048  EPI_ISL_839049  EPI_ISL_839052  EPI_ISL_839057  EPI_ISL_839058  EPI_ISL_839059  EPI_ISL_839061  EPI_ISL_839062  EPI_ISL_839063  EPI_ISL_839064  EPI_ISL_839067  EPI_ISL_839069  EPI_ISL_839072  EPI_ISL_839075  EPI_ISL_839077  EPI_ISL_839084  EPI_ISL_839085  EPI_ISL_839087  EPI_ISL_839089  EPI_ISL_839091  EPI_ISL_839097  EPI_ISL_839098  EPI_ISL_839099  EPI_ISL_839100  EPI_ISL_839106  EPI_ISL_839107  EPI_ISL_839120  EPI_ISL_839122  EPI_ISL_839125  EPI_ISL_839126  EPI_ISL_839129  EPI_ISL_839131  EPI_ISL_839140  EPI_ISL_839141  EPI_ISL_839142  EPI_ISL_839144  EPI_ISL_839148  EPI_ISL_839151  EPI_ISL_839152  EPI_ISL_839156  EPI_ISL_839161  EPI_ISL_839163  EPI_ISL_839166  EPI_ISL_839167  EPI_ISL_839169  EPI_ISL_839171  EPI_ISL_839174  EPI_ISL_839176  EPI_ISL_839179  EPI_ISL_839183  EPI_ISL_839189  EPI_ISL_839190  EPI_ISL_839198  EPI_ISL_839200  EPI_ISL_839204  EPI_ISL_839209  EPI_ISL_839211  EPI_ISL_839214  EPI_ISL_839224  EPI_ISL_839225  EPI_ISL_839227  EPI_ISL_839229  EPI_ISL_839231  EPI_ISL_839233  EPI_ISL_839235  EPI_ISL_839236  EPI_ISL_839237  EPI_ISL_839238  EPI_ISL_839244  EPI_ISL_839249  EPI_ISL_839252  EPI_ISL_839253  EPI_ISL_839255  EPI_ISL_839256  EPI_ISL_839257  EPI_ISL_839258  EPI_ISL_839261  EPI_ISL_839267  EPI_ISL_839271  EPI_ISL_839272  EPI_ISL_839274  EPI_ISL_839275  EPI_ISL_839276  EPI_ISL_839277  EPI_ISL_839283  EPI_ISL_839284  EPI_ISL_839286  EPI_ISL_839287  EPI_ISL_839289  EPI_ISL_839292  EPI_ISL_839296  EPI_ISL_839299  EPI_ISL_839301  EPI_ISL_839302  EPI_ISL_865893  EPI_ISL_865897  EPI_ISL_865904  EPI_ISL_865908  EPI_ISL_865909  EPI_ISL_865910  EPI_ISL_865912  EPI_ISL_865913  EPI_ISL_865914  EPI_ISL_865915  EPI_ISL_865918  EPI_ISL_865919  EPI_ISL_865920  EPI_ISL_865926  EPI_ISL_865928  EPI_ISL_865931  EPI_ISL_865932  EPI_ISL_865934  EPI_ISL_865935  EPI_ISL_865937  EPI_ISL_865940  EPI_ISL_865941  EPI_ISL_865943  EPI_ISL_865945  EPI_ISL_865947  EPI_ISL_865951  EPI_ISL_865954  EPI_ISL_865958  EPI_ISL_865960  EPI_ISL_865961  EPI_ISL_865965  EPI_ISL_865967  EPI_ISL_865968  EPI_ISL_865977  EPI_ISL_865980  EPI_ISL_865981  EPI_ISL_865983  EPI_ISL_865984  EPI_ISL_865985  EPI_ISL_865987  EPI_ISL_865991  EPI_ISL_865994  EPI_ISL_865995  EPI_ISL_866000  EPI_ISL_866007  EPI_ISL_866008  EPI_ISL_866009  EPI_ISL_866012  EPI_ISL_866017  EPI_ISL_866019  EPI_ISL_866024  EPI_ISL_866026  EPI_ISL_866028  EPI_ISL_866029  EPI_ISL_866031  EPI_ISL_866034  EPI_ISL_866036  EPI_ISL_866037  EPI_ISL_866041  EPI_ISL_866046  EPI_ISL_920098  EPI_ISL_920099  EPI_ISL_920102  EPI_ISL_920107  EPI_ISL_920112  EPI_ISL_920114  EPI_ISL_920116  EPI_ISL_920118  EPI_ISL_920125  EPI_ISL_920126  EPI_ISL_920128  EPI_ISL_920130  EPI_ISL_920133  EPI_ISL_920135  EPI_ISL_920139  EPI_ISL_920140  EPI_ISL_920141  EPI_ISL_920143  EPI_ISL_920145  EPI_ISL_920147  EPI_ISL_920149  EPI_ISL_920153  EPI_ISL_920154  EPI_ISL_920155  EPI_ISL_920156  EPI_ISL_920158  EPI_ISL_920159  EPI_ISL_920163  EPI_ISL_920166  EPI_ISL_920167  EPI_ISL_920173  EPI_ISL_949917  EPI_ISL_949923  EPI_ISL_949926  EPI_ISL_949929  EPI_ISL_949932  EPI_ISL_949934  EPI_ISL_949939  EPI_ISL_949940  EPI_ISL_949943  EPI_ISL_949945  EPI_ISL_949946  EPI_ISL_949947  EPI_ISL_949949  EPI_ISL_949951  EPI_ISL_949954  EPI_ISL_949961  EPI_ISL_949966  EPI_ISL_949970  EPI_ISL_949972  EPI_ISL_997843  EPI_ISL_997845  EPI_ISL_997846  EPI_ISL_997848  EPI_ISL_997850  EPI_ISL_997851  EPI_ISL_997853  EPI_ISL_997855  EPI_ISL_997862  EPI_ISL_997864  EPI_ISL_997870  EPI_ISL_997876 | hCoV-19/England/LOND-12F53F1/2021  hCoV-19/England/LOND-12F543A/2021  hCoV-19/England/LOND-12F5449/2021  hCoV-19/England/LOND-12F5458/2021  hCoV-19/England/LOND-12F5485/2021  hCoV-19/England/LOND-12F54B2/2021  hCoV-19/England/LOND-12F54C1/2021  hCoV-19/England/LOND-12F550A/2021  hCoV-19/England/LOND-12F5573/2021  hCoV-19/England/LOND-12F55DD/2021  hCoV-19/England/LOND-12F5652/2021  hCoV-19/England/LOND-12F568F/2021  hCoV-19/England/LOND-12F569E/2021  hCoV-19/England/LOND-12F56AD/2021  hCoV-19/England/LOND-12F56CB/2021  hCoV-19/England/LOND-12F56F8/2021  hCoV-19/England/LOND-12F5731/2021  hCoV-19/England/LOND-12F576E/2021  hCoV-19/England/LOND-12F57F5/2021  hCoV-19/England/LOND-12F5810/2021  hCoV-19/England/LOND-12F582F/2021  hCoV-19/England/LOND-12F583E/2021  hCoV-19/England/LOND-12F5A92/2021  hCoV-19/England/LOND-12F5B26/2021  hCoV-19/England/LOND-12F5B44/2021  hCoV-19/England/LOND-12F5B80/2021  hCoV-19/England/LOND-12F0F89/2020  hCoV-19/England/LOND-12F1119/2020  hCoV-19/England/LOND-12F1164/2020  hCoV-19/England/LOND-12F1182/2020  hCoV-19/England/LOND-12F11BF/2020  hCoV-19/England/LOND-12F129E/2020  hCoV-19/England/LOND-12F7E58/2021  hCoV-19/England/LOND-12F7E76/2021  hCoV-19/England/LOND-12F7E85/2021  hCoV-19/England/LOND-12F81C7/2021  hCoV-19/England/LOND-12F8482/2021  hCoV-19/England/LOND-12F859E/2021  hCoV-19/England/LOND-12F86C8/2021  hCoV-19/England/LOND-12F8947/2021  hCoV-19/England/LOND-12F8A71/2021  hCoV-19/England/LOND-12F8AAE/2021  hCoV-19/England/LOND-12F8ADB/2021  hCoV-19/England/LOND-12F6D10/2021  hCoV-19/England/LOND-12F82F1/2021  hCoV-19/England/LOND-12F87D4/2021  hCoV-19/England/LOND-12F8956/2021  hCoV-19/England/LOND-12F8A62/2021  hCoV-19/England/LOND-12F8A9F/2021  hCoV-19/England/LOND-12F8BAB/2021  hCoV-19/England/LOND-12F35A2/2021  hCoV-19/England/LOND-12F36FA/2020  hCoV-19/England/LOND-12F3706/2020  hCoV-19/England/LOND-12F3CE9/2020  hCoV-19/England/LOND-12F4064/2021  hCoV-19/England/LOND-12F4152/2021  hCoV-19/England/LOND-12F4204/2021  hCoV-19/England/LOND-12F4486/2021  hCoV-19/England/LOND-12F44D1/2021  hCoV-19/England/LOND-12F4565/2021  hCoV-19/England/LOND-12F45FC/2021  hCoV-19/England/LOND-12F4626/2021  hCoV-19/England/LOND-12F4B81/2021  hCoV-19/England/LOND-12F4D03/2021  hCoV-19/England/LOND-12F4D21/2021  hCoV-19/England/LOND-12F4D4F/2021  hCoV-19/England/LOND-12F4D9A/2021  hCoV-19/England/LOND-12F4E00/2021  hCoV-19/England/LOND-12F4EE2/2021  hCoV-19/England/LOND-12F4EF1/2021  hCoV-19/England/LOND-12F5607/2021  hCoV-19/England/LOND-12F56BC/2021  hCoV-19/England/LOND-12F59D1/2021  hCoV-19/England/LOND-12F5A1A/2021  hCoV-19/England/LOND-12F5A29/2021  hCoV-19/England/LOND-12F5A38/2021  hCoV-19/England/LOND-12F5DC6/2021  hCoV-19/England/LOND-12F6062/2021  hCoV-19/England/LOND-12F6080/2021  hCoV-19/England/LOND-12F6396/2021  hCoV-19/England/LOND-12F63F0/2021  hCoV-19/England/LOND-12F66AC/2021  hCoV-19/England/LOND-12F682E/2021  hCoV-19/England/LOND-12F6879/2021  hCoV-19/England/LOND-12F6888/2021  hCoV-19/England/LOND-12F6897/2021  hCoV-19/England/LOND-12F68D3/2021  hCoV-19/England/LOND-12F6994/2021  hCoV-19/England/LOND-12F6AA0/2021  hCoV-19/England/LOND-12F6BCB/2021  hCoV-19/England/LOND-12F6CAA/2021  hCoV-19/England/LOND-12F6D89/2021  hCoV-19/England/LOND-12F6E2C/2021  hCoV-19/England/LOND-12F6E59/2021  hCoV-19/England/LOND-12F725C/2021  hCoV-19/England/LOND-12F73A4/2021  hCoV-19/England/LOND-12F73C2/2021  hCoV-19/England/LOND-12F7483/2021  hCoV-19/England/LOND-12F74FC/2021  hCoV-19/England/LOND-12F77D5/2021  hCoV-19/England/LOND-12F78D2/2021  hCoV-19/England/LOND-12F7957/2021  hCoV-19/England/LOND-12F79B1/2021  hCoV-19/England/LOND-12F7BF7/2021  hCoV-19/England/LOND-12F8033/2021  hCoV-19/England/LOND-12F82A6/2021  hCoV-19/England/LOND-12F8419/2021  hCoV-19/England/LOND-12F8516/2021  hCoV-19/England/LOND-12F87E3/2021  hCoV-19/England/LOND-12F8B9C/2021  hCoV-19/England/LOND-12F2D50/2020  hCoV-19/England/LOND-12F33C6/2020  hCoV-19/England/LOND-12F3AFE/2021  hCoV-19/England/LOND-12F3BDD/2021  hCoV-19/England/LOND-12F3C52/2021  hCoV-19/England/LOND-12F3C61/2021  hCoV-19/England/LOND-12F3C70/2021  hCoV-19/England/LOND-12F3CAD/2020  hCoV-19/England/LOND-12F3D9B/2021  hCoV-19/England/LOND-12F2109/2020  hCoV-19/England/LOND-12F0129/2020  hCoV-19/England/LOND-12F03E7/2020  hCoV-19/England/LOND-12F07CD/2020  hCoV-19/England/LOND-12F0806/2020  hCoV-19/England/LOND-12F0824/2020  hCoV-19/England/LOND-12F08AC/2020  hCoV-19/England/LOND-12F08CA/2020  hCoV-19/England/LOND-12F094F/2020  hCoV-19/England/LOND-12F09A9/2020  hCoV-19/England/LOND-12F0AC4/2020  hCoV-19/England/LOND-12F0AE2/2020  hCoV-19/England/LOND-12F0B3A/2020  hCoV-19/England/LOND-12F0BA3/2020  hCoV-19/England/LOND-12F0BC1/2020  hCoV-19/England/LOND-12F0C19/2020  hCoV-19/England/LOND-12F0C28/2020  hCoV-19/England/LOND-12F0C55/2020  hCoV-19/England/LOND-12F0CCE/2020  hCoV-19/England/LOND-12F0D25/2020  hCoV-19/England/LOND-12F0D34/2020  hCoV-19/England/LOND-12F0D16/2020  hCoV-19/England/LOND-12F0F5C/2020  hCoV-19/England/LOND-12F0E6E/2020  hCoV-19/England/LOND-12F0DF8/2020  hCoV-19/England/LOND-12F0EE6/2020  hCoV-19/England/LOND-12F0F3E/2020  hCoV-19/England/LOND-12F10A3/2020  hCoV-19/England/LOND-12F10C1/2020  hCoV-19/England/LOND-12F135F/2020  hCoV-19/England/LOND-12F1692/2020  hCoV-19/England/LOND-12F16A1/2020  hCoV-19/England/LOND-12F143E/2020  hCoV-19/England/LOND-12F18BA/2020  hCoV-19/England/LOND-12F1832/2020  hCoV-19/England/LOND-12F1920/2020  hCoV-19/England/LOND-12F1735/2020  hCoV-19/England/LOND-12F16ED/2020  hCoV-19/England/LOND-12F1814/2020  hCoV-19/England/LOND-12F1665/2020  hCoV-19/England/LOND-12F15FF/2020  hCoV-19/England/LOND-12F15D1/2020  hCoV-19/England/LOND-12F1762/2020  hCoV-19/England/LOND-12F1780/2020  hCoV-19/England/LOND-12F186F/2020  hCoV-19/England/LOND-12F189C/2020  hCoV-19/England/LOND-12F1AB4/2020  hCoV-19/England/LOND-12F1683/2020  hCoV-19/England/LOND-12F16CF/2020  hCoV-19/England/LOND-12F1AF0/2020  hCoV-19/England/LOND-12F161A/2020  hCoV-19/England/LOND-12F1647/2020  hCoV-19/England/LOND-12F19F3/2020  hCoV-19/England/LOND-12F1A0F/2020  hCoV-19/England/LOND-12F1B57/2020  hCoV-19/England/LOND-12F1B66/2020  hCoV-19/England/LOND-12F1B93/2020  hCoV-19/England/LOND-12F1D9D/2020  hCoV-19/England/LOND-12F1DAC/2020  hCoV-19/England/LOND-12F1E6D/2020  hCoV-19/England/LOND-12F1E7C/2020  hCoV-19/England/LOND-12F1E8B/2020  hCoV-19/England/LOND-12F1E9A/2020  hCoV-19/England/LOND-12F1EA9/2020  hCoV-19/England/LOND-12F1ED6/2020  hCoV-19/England/LOND-12F1EE5/2020  hCoV-19/England/LOND-12F1F00/2020  hCoV-19/England/LOND-12F1F4C/2020  hCoV-19/England/LOND-12F1F5B/2020  hCoV-19/England/LOND-12F1F6A/2020  hCoV-19/England/LOND-12F1F79/2020  hCoV-19/England/LOND-12F202A/2020  hCoV-19/England/LOND-12F2206/2020  hCoV-19/England/LOND-12F2172/2020  hCoV-19/England/LOND-12F2400/2020  hCoV-19/England/LOND-12F2F69/2020  hCoV-19/England/LOND-12F228E/2020  hCoV-19/England/LOND-12F2488/2020  hCoV-19/England/LOND-12F2233/2020  hCoV-19/England/LOND-12F22F7/2020  hCoV-19/England/LOND-12F31CC/2020  hCoV-19/England/LOND-12F24F1/2020  hCoV-19/England/LOND-12F2260/2020  hCoV-19/England/LOND-12F2ED5/2020  hCoV-19/England/LOND-12F241F/2020  hCoV-19/England/LOND-12F2576/2020  hCoV-19/England/LOND-12F3056/2020  hCoV-19/England/LOND-12F2673/2020  hCoV-19/England/LOND-12F2619/2020  hCoV-19/England/LOND-12F26DD/2020  hCoV-19/England/LOND-12F2F0F/2020  hCoV-19/England/LOND-12F2655/2020  hCoV-19/England/LOND-12F2628/2020  hCoV-19/England/LOND-12F2691/2020  hCoV-19/England/LOND-12F2FA5/2020  hCoV-19/England/LOND-12F3047/2020  hCoV-19/England/LOND-12F287D/2020  hCoV-19/England/LOND-12F2831/2020  hCoV-19/England/LOND-12F2716/2020  hCoV-19/England/LOND-12F2A86/2020  hCoV-19/England/LOND-12F28AA/2020  hCoV-19/England/LOND-12F2B38/2020  hCoV-19/England/LOND-12F2075/2020  hCoV-19/England/LOND-12F2B74/2020  hCoV-19/England/LOND-12F2989/2020  hCoV-19/England/LOND-12F20A2/2020  hCoV-19/England/LOND-12F2F78/2020  hCoV-19/England/LOND-12F2B56/2020  hCoV-19/England/LOND-12F2BB0/2020  hCoV-19/England/LOND-12F3083/2020  hCoV-19/England/LOND-12F2910/2020  hCoV-19/England/LOND-12F2BCF/2020  hCoV-19/England/LOND-12F293E/2020  hCoV-19/England/LOND-12F2C62/2020  hCoV-19/England/LOND-12F2CDB/2020  hCoV-19/England/LOND-12F2AA4/2020  hCoV-19/England/LOND-12F2CEA/2020  hCoV-19/England/LOND-12F3074/2020  hCoV-19/England/LOND-12F297A/2020  hCoV-19/England/LOND-12F30CF/2020  hCoV-19/England/LOND-12F2901/2020  hCoV-19/England/LOND-12F32C9/2020  hCoV-19/England/LOND-12F30FC/2020  hCoV-19/England/LOND-12F2C17/2020  hCoV-19/England/LOND-12F2EE4/2020  hCoV-19/England/LOND-12F2A1D/2020  hCoV-19/England/LOND-12F2BA1/2020  hCoV-19/England/LOND-12F3241/2020  hCoV-19/England/LOND-12F294D/2020  hCoV-19/England/LOND-12F32E7/2020  hCoV-19/England/LOND-12F3214/2020  hCoV-19/England/LOND-12F289B/2020  hCoV-19/England/LOND-12F2D14/2021  hCoV-19/England/LOND-12F334E/2020  hCoV-19/England/LOND-12F335D/2020  hCoV-19/England/LOND-12F34F0/2021  hCoV-19/England/LOND-12F3566/2021  hCoV-19/England/LOND-12F3575/2021  hCoV-19/England/LOND-12F35B1/2021  hCoV-19/England/LOND-12F3609/2021  hCoV-19/England/LOND-12F3618/2021  hCoV-19/England/LOND-12F3627/2021  hCoV-19/England/LOND-12F3654/2021  hCoV-19/England/LOND-12F3663/2021  hCoV-19/England/LOND-12F3681/2020  hCoV-19/England/LOND-12F3715/2020  hCoV-19/England/LOND-12F3760/2020  hCoV-19/England/LOND-12F378E/2020  hCoV-19/England/LOND-12F37BB/2021  hCoV-19/England/LOND-12F37E8/2021  hCoV-19/England/LOND-12F3821/2021  hCoV-19/England/LOND-12F38D6/2020  hCoV-19/England/LOND-12F38E5/2021  hCoV-19/England/LOND-12F393D/2021  hCoV-19/England/LOND-12F395B/2021  hCoV-19/England/LOND-12F3979/2021  hCoV-19/England/LOND-12F39E2/2021  hCoV-19/England/LOND-12F39F1/2021  hCoV-19/England/LOND-12F3A1C/2021  hCoV-19/England/LOND-12F3A2B/2021  hCoV-19/England/LOND-12F3EE3/2020  hCoV-19/England/LOND-12F3EF2/2020  hCoV-19/England/LOND-12F4019/2021  hCoV-19/England/LOND-12F4073/2021  hCoV-19/England/LOND-12F40BF/2021  hCoV-19/England/LOND-12F40DD/2021  hCoV-19/England/LOND-12F4116/2021  hCoV-19/England/LOND-12F4134/2021  hCoV-19/England/LOND-12F41F8/2021  hCoV-19/England/LOND-12F4213/2021  hCoV-19/England/LOND-12F4222/2021  hCoV-19/England/LOND-12F425F/2021  hCoV-19/England/LOND-12F429B/2021  hCoV-19/England/LOND-12F42C8/2021  hCoV-19/England/LOND-12F42D7/2021  hCoV-19/England/LOND-12F432F/2021  hCoV-19/England/LOND-12F437A/2021  hCoV-19/England/LOND-12F4398/2021  hCoV-19/England/LOND-12F43D4/2021  hCoV-19/England/LOND-12F43E3/2021  hCoV-19/England/LOND-12F4547/2021  hCoV-19/England/LOND-12F4583/2021  hCoV-19/England/LOND-12F46F9/2021  hCoV-19/England/LOND-12F4714/2021  hCoV-19/England/LOND-12F4750/2021  hCoV-19/England/LOND-12F47AB/2021  hCoV-19/England/LOND-12F4802/2021  hCoV-19/England/LOND-12F4811/2021  hCoV-19/England/LOND-12F4899/2021  hCoV-19/England/LOND-12F48B7/2021  hCoV-19/England/LOND-12F491E/2021  hCoV-19/England/LOND-12F4969/2021  hCoV-19/England/LOND-12F4987/2021  hCoV-19/England/LOND-12F49D2/2021  hCoV-19/England/LOND-12F4A93/2021  hCoV-19/England/LOND-12F4AA2/2021  hCoV-19/England/LOND-12F4AC0/2020  hCoV-19/England/LOND-12F4AFD/2021  hCoV-19/England/LOND-12F4BAF/2021  hCoV-19/England/LOND-12F4BDC/2021  hCoV-19/England/LOND-12F4C06/2021  hCoV-19/England/LOND-12F4C15/2021  hCoV-19/England/LOND-12F4C24/2021  hCoV-19/England/LOND-12F4C33/2021  hCoV-19/England/LOND-12F4CAC/2021  hCoV-19/England/LOND-12F4EC4/2021  hCoV-19/England/LOND-12F4F49/2021  hCoV-19/England/LOND-12F4F76/2021  hCoV-19/England/LOND-12F4FA3/2021  hCoV-19/England/LOND-12F4FB2/2021  hCoV-19/England/LOND-12F4FC1/2021  hCoV-19/England/LOND-12F4FD0/2021  hCoV-19/England/LOND-12F5009/2021  hCoV-19/England/LOND-12F50AF/2021  hCoV-19/England/LOND-12F50EB/2021  hCoV-19/England/LOND-12F50FA/2021  hCoV-19/England/LOND-12F5115/2021  hCoV-19/England/LOND-12F5124/2021  hCoV-19/England/LOND-12F5151/2021  hCoV-19/England/LOND-12F517F/2021  hCoV-19/England/LOND-12F5212/2021  hCoV-19/England/LOND-12F5230/2021  hCoV-19/England/LOND-12F525E/2021  hCoV-19/England/LOND-12F526D/2021  hCoV-19/England/LOND-12F528B/2021  hCoV-19/England/LOND-12F52B8/2021  hCoV-19/England/LOND-12F52F4/2021  hCoV-19/England/LOND-12F532E/2021  hCoV-19/England/LOND-12F534C/2021  hCoV-19/England/LOND-12F535B/2021  hCoV-19/England/LOND-12F584D/2020  hCoV-19/England/LOND-12F58D4/2021  hCoV-19/England/LOND-12F5C50/2021  hCoV-19/England/LOND-12F5CC9/2021  hCoV-19/England/LOND-12F5CD8/2021  hCoV-19/England/LOND-12F5CE7/2021  hCoV-19/England/LOND-12F5D20/2021  hCoV-19/England/LOND-12F5D3F/2021  hCoV-19/England/LOND-12F5D4E/2021  hCoV-19/England/LOND-12F5D5D/2021  hCoV-19/England/LOND-12F5D8A/2021  hCoV-19/England/LOND-12F5DA8/2021  hCoV-19/England/LOND-12F5DE4/2021  hCoV-19/England/LOND-12F5EF0/2021  hCoV-19/England/LOND-12F5F1B/2021  hCoV-19/England/LOND-12F5F93/2021  hCoV-19/England/LOND-12F5FA2/2021  hCoV-19/England/LOND-12F5FC0/2021  hCoV-19/England/LOND-12F6017/2021  hCoV-19/England/LOND-12F6035/2021  hCoV-19/England/LOND-12F609F/2021  hCoV-19/England/LOND-12F60AE/2021  hCoV-19/England/LOND-12F60CC/2021  hCoV-19/England/LOND-12F6105/2021  hCoV-19/England/LOND-12F6123/2021  hCoV-19/England/LOND-12F618D/2021  hCoV-19/England/LOND-12F61D8/2021  hCoV-19/England/LOND-12F625D/2021  hCoV-19/England/LOND-12F628A/2021  hCoV-19/England/LOND-12F62B7/2021  hCoV-19/England/LOND-12F630F/2021  hCoV-19/England/LOND-12F6369/2021  hCoV-19/England/LOND-12F6387/2021  hCoV-19/England/LOND-12F6536/2021  hCoV-19/England/LOND-12F65BE/2021  hCoV-19/England/LOND-12F65CD/2021  hCoV-19/England/LOND-12F65EB/2021  hCoV-19/England/LOND-12F65FA/2021  hCoV-19/England/LOND-12F66F7/2021  hCoV-19/England/LOND-12F6712/2021  hCoV-19/England/LOND-12F679A/2021  hCoV-19/England/LOND-12F685B/2021  hCoV-19/England/LOND-12F686A/2021  hCoV-19/England/LOND-12F69C1/2021  hCoV-19/England/LOND-12F6B34/2021  hCoV-19/England/LOND-12F6B8F/2021  hCoV-19/England/LOND-12F6BAD/2021  hCoV-19/England/LOND-12F6CF5/2021  hCoV-19/England/LOND-12F6DC5/2021  hCoV-19/England/LOND-12F6F47/2021  hCoV-19/England/LOND-12F774E/2021  hCoV-19/England/LOND-12F778A/2021  hCoV-19/England/LOND-12F77A8/2021  hCoV-19/England/LOND-12F77B7/2021  hCoV-19/England/LOND-12F782D/2021  hCoV-19/England/LOND-12F785A/2021  hCoV-19/England/LOND-12F7878/2021  hCoV-19/England/LOND-12F7887/2021  hCoV-19/England/LOND-12F78C3/2021  hCoV-19/England/LOND-12F7966/2021  hCoV-19/England/LOND-12F7070/2021  hCoV-19/England/LOND-12F708F/2021  hCoV-19/England/LOND-12F70DA/2021  hCoV-19/England/LOND-12F7131/2021  hCoV-19/England/LOND-12F719B/2021  hCoV-19/England/LOND-12F71C8/2021  hCoV-19/England/LOND-12F71E6/2021  hCoV-19/England/LOND-12F7298/2021  hCoV-19/England/LOND-12F732C/2021  hCoV-19/England/LOND-12F733B/2021  hCoV-19/England/LOND-12F7474/2021  hCoV-19/England/LOND-12F74DE/2021  hCoV-19/England/LOND-12F7526/2021  hCoV-19/England/LOND-12F7553/2021  hCoV-19/England/LOND-12F759F/2021  hCoV-19/England/LOND-12F75BD/2021  hCoV-19/England/LOND-12F75CC/2021  hCoV-19/England/LOND-12F75EA/2021  hCoV-19/England/LOND-12F7605/2021  hCoV-19/England/LOND-12F7623/2021  hCoV-19/England/LOND-12F7650/2021  hCoV-19/England/LOND-12F76D8/2021  hCoV-19/England/LOND-12F76E7/2021  hCoV-19/England/LOND-12F775D/2021  hCoV-19/England/LOND-12F777B/2021  hCoV-19/England/LOND-12F79EE/2021  hCoV-19/England/LOND-12F7A27/2021  hCoV-19/England/LOND-12F7A72/2021  hCoV-19/England/LOND-12F7ABE/2021  hCoV-19/England/LOND-12F7ACD/2021  hCoV-19/England/LOND-12F7B7F/2021  hCoV-19/England/LOND-12F6475/2021  hCoV-19/England/LOND-12F64EE/2021  hCoV-19/England/LOND-12F668E/2021  hCoV-19/England/LOND-12F67C7/2021  hCoV-19/England/LOND-12F692B/2021  hCoV-19/England/LOND-12F6967/2021  hCoV-19/England/LOND-12F6B07/2021  hCoV-19/England/LOND-12F6B16/2021  hCoV-19/England/LOND-12F6C40/2021  hCoV-19/England/LOND-12F6C9B/2021  hCoV-19/England/LOND-12F6CB9/2021  hCoV-19/England/LOND-12F6CE6/2021  hCoV-19/England/LOND-12F6DE3/2021  hCoV-19/England/LOND-12F6E0E/2021  hCoV-19/England/LOND-12F6E4A/2021  hCoV-19/England/LOND-12F6EFF/2021  hCoV-19/England/LOND-12F7C7C/2021  hCoV-19/England/LOND-12F7CC7/2021  hCoV-19/England/LOND-12F7CF4/2021  hCoV-19/England/LOND-12F7C4F/2021  hCoV-19/England/LOND-12F7D3D/2021  hCoV-19/England/LOND-12F7D4C/2021  hCoV-19/England/LOND-12F7D79/2021  hCoV-19/England/LOND-12F7D97/2021  hCoV-19/England/LOND-12F7DA6/2021  hCoV-19/England/LOND-12F7DC4/2021  hCoV-19/England/LOND-12F7E1C/2021  hCoV-19/England/LOND-12F7FA0/2021  hCoV-19/England/LOND-12F7FDD/2021  hCoV-19/England/LOND-12F809D/2021  hCoV-19/England/LOND-12F815E/2021 |
