# Supplementary material for: Post-vaccination COVID-19: A case-control study and genomic analysis of 119 breakthrough infections in partially vaccinated individuals
Source: Clin Infect Dis. 2021 Aug 19:ciab714. doi: 10.1093/cid/ciab714 (PMC8513403; doi:10.1093/cid/ciab714)
Supplement: Supplementary file 1 [file ciab714_suppl_Supplementary_Materials.docx]

Protocol

This protocol has been provided by the authors to give readers additional information about their work.

**Rationale for this study**

Post-vaccination infections constitute an important threat to the successful control of the COVID-19 pandemic by current available vaccines. Viral replication during a non-sterilizing immune response may favor vaccine-escaping mutations. Additionally, apart from post-vaccination COVID-19 infection rates, there is paucity of data on patient-oriented outcomes, including death, after vaccination. Real-world data evaluating vaccine effectiveness is urgently needed[1].

**Study objectives**

This study aimed to:

Evaluate vaccine effectiveness against patient-oriented outcomes in a real-world setting, and investigate if SARS-CoV-2 infection after vaccination is associated with viral genetic changes.

The specific objectives were:

- Determine whether previous vaccination reduces all-cause death by 60 days after the patient’s 1st SARS-COV-2 positive sample.

- Determine whether previous vaccination reduces probability of admission to hospital within 14 days after the patient’s 1st SARS-COV-2 positive sample.

- Determine whether previous vaccination reduces length of stay in hospital in patients who eventually get hospitalized within 14 days after the patient’s1st SARS-COV-2 positive sample.

- Determine whether previous vaccination increases the cycle threshold value (Ct) of documented infections compared to infections from non-vaccinated individuals.

- Evaluate whether previous vaccination is associated with measurable changes in SARS-CoV-2 genome, including mutations, single nucleotide polymorphisms (SNPs) and minority variant alleles (MVAs).

- Compare the phylogenies of SARS-CoV-2 strains isolated from vaccinated patients to the ones isolated from non-vaccinated patients.

**Methods**

**Setting**

The Royal Free Hospital is a tertiary teaching hospital in central London, with an 830 inpatient bed capacity, sharing a catchment area of 2.5 million people with two district general hospitals. It offers general and specialist services, including emergency, maternity, infectious diseases, cardiothoracic surgery, solid organ transplantation, kidney dialysis, and is a national referral centre for amyloidosis, haemophilia, lysosomal storage disorders and neuroendocrine tumors. During the COVID-19 pandemic, the hospital escalated its Level 3 ventilated bed capacity to more than 100 beds.

The Royal Free Hospital is a collaborator of the COVID-19 Genomics UK Consortium (COG-UK), a UK publicly funded partnership of public health agencies, academic partners, diagnostic laboratories and NHS organizations, which performs decentralized full-genome sequencing of clinical COVID-19 samples. The Consortium was established in April 2020, and has thus far sequenced more than 450.000 clinical samples by May 2021. The Royal Free Hospital is also a study site for the COG-UK Hospital-Onset COVID-19 Infections (HOCI) Study, a National Institute for Health Research (NIHR) Urgent Public Health-Badged Clinical Study, which aims to investigate the molecular epidemiology of SARS-CoV-2 transmission within healthcare settings.  Our study was nested within the COG-UK HOCI study.

**Ethics**

Ethical approval for the COG-UK HOCI study was provided by the NHS Integrated Research Application System (IRAS) Research Ethics Committee (REC) 20/EE/0118. The University College London DNA Infection Bank Research Ethics Committee waived the need for participant informed consent for this study (Ref 17/LO/1530).

**Study Design**

This protocol describes a retrospective case-control study. All post-vaccination COVID-19 positive participants (cases) identified within the study time period were randomly age (±3 years) and sex matched to eligible participants with COVID-19 infection within the same period, and from the same institution (controls). The matching ratio was determined by the number of controls eligible for matching, and continued until the minimum ratio of cases to controls was reached. This means that if an additional round of matching occurred, at least one case will have no additional eligible matching controls. This has been shown to maximize statistical power when the number of cases is predetermined[2]. Each control was eligible for matching with one case only. 

**Study population and data time period**

Eligible participants were identified through SARS-CoV-2 positive samples submitted by the Royal Free Hospital to COG-UK for sequencing in the context of the COG-UK HOCI study. All positive cases between the 30th of September 2020 and the 15th of March 2021 were included. Only the first positive sample was considered from each patient, and subsequent samples were excluded, even if they were considered to represent reinfections. Positive patients, whose sample was not available for sequencing and therefore were not included into the COG-UK-HOCI study, were excluded. No other exclusion criteria applied. Follow-up of each participant continued for up 60 days after the index COVID-19 positive sample, or until their date of death, and ended at the latest on the 14th of May 2021 for all participants. During the study period, only vaccination with BNT162b2 or ChAdOx1 was available in the UK, initiating on the 8th of December 2020 and 4th of January 2021 respectively.

**Data collection**

The Royal Free Hospital uses the electronic health record Powerchart (Cerner Corporation, North Kansas City, MO), in which patients’ comorbidities are recorded via ICD-10 codes by the hospital’s clinical coding team after each patient encounter. It also contains demographics and information on patients’ admissions and length of stay in hospital. For this study, all ICD-10 codes assigned to each participant at the time of the index COVID-19 sample, or encounters before that, were automatically extracted by information analysts. Demographics, admission and discharge dates within 14 days of the index COVID-19 sample were also pulled. Prespecified comorbidities were subsequently assigned according to the extracted ICD-10 codes. For patients with zero assigned ICD-10 codes, manual inspection of their hospital electronic health record was performed, including their primary care record, if necessary. Dates of death and dates of vaccination were manually collected from the Nation Health Service (NHS) Summary Care Record, which is the UK national electronic summary of patients’ key clinical information, sourced from their GP record. To avoid confirmation bias, investigators were blinded to patient outcomes while determining patient comorbidities, and vice versa. They were also blinded to the results of the genomic analysis.

**Variable definitions**

**COVID-19**

COVID-19 infection was defined as the detection of SARS-CoV-2 RNA in a combined nose and throat swab. During the study period, the Royal Free Hospital used a combination of the following assays for diagnosis of the infection: a laboratory-developed ‘N gene’ PCR assay[3] run on the open channel of the Panther Fusion System (Hologic, Marlborough, MA),  a laboratory-developed extraction-free PCR assay[3], Aptima SARS-CoV-2 TMA assay run on the Panther System (Hologic, Marlborough, MA), GeneXpert Xpress SARSCoV-2 (Cepheid, Sunnyvale, CA), Idylla SARS-CoV-2 Test (Biocartis, Mechelen, Belgium). All samples were processed and reported according to respective manufacturer’s instructions.

**Outcome Variables**

- All-cause death, as reported in the NHS Summary Care Record, before 60 days of the index COVID-19 positive sample.

- Hospital admission, defined as an inpatient encounter for any period of time within 14 days of the index COVID-19 sample.

- Length of stay in hospital, defined as the difference in days between patient admission date and discharge date, if the index COVID-19 test preceded the admission date, or the difference between the sample date and the discharge date, if the index COVID-19 test was after the admission date. Length of stay in hospital was analyzed only for patients who survived their admission.

**Exposure variables**

- Age: Difference in years between date of index COVID-19 sample and date of birth

- Sex: Male or Female

- Ethnicity: White ethnicity included the subcategories: British, Irish, White – British, White – any other White background, Any other White background. Asian ethnicity included the following subcategories: Indian, Pakistani, Asian or Asian British: Indian, Asian or Asian British – Pakistani, Bangladeshi, Any other Asian Background, Asian – any other Asian background. Black ethnicity included the following subcategories: African, Caribbean, Black – Any other Black background, Any other black background.  Mixed/Other ethnicity included the following subcategories: White and Asian, White and Black African, White and Black Caribbean, Mixed – Any other mixed background, Any other mixed background. If ethnicity was not recorded in the hospital electronic record, that was obtained by the National Primary Care Record. 

- Multiple Index of Deprivation: Calculated by the Ministry of Housing, Communities & Local Government’s English indices of deprivation 2019 (accessed at [https://imd-by-postcode.opendatacommunities.org/imd/2019](https://imd-by-postcode.opendatacommunities.org/imd/2019)) according to patients’ postcode. Patients of no fixed abode were automatically assigned the lowest index. Patients were subsequently split into four quartiles from lowest deprivation (1st quartile) to highest (4th quartile).

- Care home or nursing home residency: As documented at the time of the index COVID-19 sample. If not available, the patient’s postcode was checked as to whether it corresponds to a UK nursing home address.

- Pregnancy: Whether the patient was pregnant at the time of the index COVID-19 sample.

- Chronic renal disease: Whether the patient had Stage IV or V chronic kidney disease at the time of the index COVID-19 sample.

- Immunosuppression: Whether the patient met the criteria for immunosuppression as defined in Chapter 6 of the PHE Green Book at the time of the index COVID-19 sample[4].

- Obesity: As defined by clinicians at the time of the index COVID-19 sample. Body mass index was not universally available for all study participants.

- Transplant: Whether the patient was a recipient of a solid organ or bone marrow transplant at the time of the index COVID-19 sample.

- Asplenia: Whether the patient had anatomic (splenectomy) or functional (sickle cell disease, splenic infraction, splenic tumor infiltration) asplenia at the time of the index COVID-19 sample.

- HIV: Whether the patient had an infection by the Human Immunodeficiency Virus 1 or 2 at the time of the index COVID-19 sample.

- Chronic respiratory disease: Whether the patient had at least one diagnosis of a chronic respiratory disease, not including asthma, at the time of the index COVID-19 sample. These diagnoses include but are not limited to: chronic obstructive pulmonary disease, emphysema, bronchiectasis, primary or secondary interstitial lung disease, pneumoconiosis, primary pulmonary hypertension, obstructive sleep apnea.

- Asthma: Whether the patient had physician-diagnosed asthma at the time of the index COVID-19 sample.

- Chronic cardiac disease: Whether the patient had at least one diagnosis of a chronic cardiac disease, not including hypertension, at the time of the index COVID-19 sample. These diagnoses include but are not limited to: ischaemic heart disease, congestive heart failure, atrial fibrillation, valvulopathies, cardiomyopathies, conduction disorders, peripheral vascular disease.

- Renal dialysis: Whether the patient was receiving renal dialysis (haemodialysis or peritoneal dialysis) at the time of the index COVID-19 sample.

- Chronic liver disease: Whether the patient had at least one diagnosis of a chronic liver disease, at the time of the index COVID-19 sample. These diagnoses include but are not limited to: cirrhosis of the liver, alcoholic liver disease, fatty liver disease, autoimmune hepatitis, primary biliary cirrhosis, sclerosing cholangitis, chronic active hepatitis B or C.

- Diabetes: Whether the patient was diagnosed with diabetes mellitus (any type) at the time of the index COVID-19 sample.

- Chronic neurological disease: Whether the patient had at least one diagnosis of a chronic neurological disease at the time of the index COVID-19 sample. These diagnoses include but are not limited to: cerebrovascular disease, Parkinson’s disease, motor neuron disease, multiple sclerosis, epilepsy, myasthenia gravis, muscular dystrophies, traumatic or anoxic brain injury, paraplegia, hydrocephalus, degenerative neurological diseases, hereditary neurological diseases, metabolic encephalopathies. 

- Active solid organ malignancy: Whether the patient had an active solid organ malignancy at the time of the index COVID-19 sample, or was on active follow up after treatment for a solid organ malignancy.

- Haematological disease: Whether the patient had at least one diagnosis of a haematological disease at the time of the index COVID-19 sample. These diagnoses include but are not limited to: lymphomas, leukemias, multiple myeloma, myelodysplastic syndrome, myelofibrosis, sarcoidosis, haemoglobinopathies, polycythaemia vera, autoimmune haemolytic anaemia, idiopathic thrombocytopenia, aplastic anaemia, agranulocytosis, haemophilia, immunoglobulin deficiencies.

- Rheumatological disease: Whether the patient had at least one diagnosis of a chronic rheumatological disease, excluding crystal arthropathies, at the time of the index COVID-19 sample. These diagnoses include but are not limited to: rheumatoid arthritis, systemic lupus erythematosus, vasculitis, Crohn’s disease, ulcerative colitis, scleroderma, psoriasis, amyloidosis, Sjogren’s syndrome, polymyalgia rheumatica, ankylosing spondylitis, fibromyalgia, glomerulonephritis.

- Dementia: Whether the patient had a diagnosis of dementia at the time of the index COVID-19 sample.

- Cycle threshold value (Ct): The Ct value as reported by the platform in which the index COVID-19 sample was processed. When two values were given (e.g., Idylla SARS-CoV-2 Biocartis platform), the lowest one was selected. For samples processed in the Aptima SARS-CoV-2 (Panther System, Hologic) platform Relative Light Units (RLU) values were obtained, which were not used for further analysis.

- Lineage: Whether the patient had a B.1.1.7 COVID-19 lineage or any other, as detected by genomic sequencing.

**Statistical analysis plan**

Statistical analysis was performed in SPSS Version 27 (IBM Corp). Initially, all variables collected were plotted, and descriptive statistics performed to detect potential outliers.

Matching was performed as previously described. After matching, cases and controls were univariably compared using the following tests:

- Mann Whitney U test: Age, Length of stay in hospital, Ct value, Number of MVAs.

- Fischer’s exact test: Death, Hospital admission, Sex, Care home or nursing home residency, Pregnancy, Chronic renal disease, Immunosuppression, Obesity, Transplant, Asplenia, HIV, Chronic respiratory disease, Asthma, Chronic cardiac disease, Renal Dialysis, Chronic liver disease, Diabetes, Chronic neurological disease, Active solid organ malignancy, Haematological disease, Rheumatological disease, Dementia, lineage.

- Pearson’s Chi squared: Age in subgroups, Ethnicity, Multiple Index of Deprivation Quartile.

Differences in risk of death between cases and controls were evaluated using multivariable Cox regression, differences in odds of admission to hospital using multivariable logistic regression, and differences in length of stay in hospital using liner regression. In each analysis, univariable differences of 0∙2 or less in confounding variables between cases and controls were considered acceptable, otherwise they were inserted into the multivariable model to control for confounding[5]. Vaccine effectiveness estimates were reported as (1 – Hazard ratio) × 100 for mortality, odds ratios for admission to hospital and difference in days for length of stay in hospital. The level of statistical significance was set at 0.05.

Prespecified subgroup analysis was performed for patients with onset of COVID-19 infection ≥14 days after vaccination, and by type of vaccination. Matching was not repeated for subgroup analysis. Instead, the same controls matched during the analysis of the entire cohort were used. No sensitivity analysis was required, as there was no missing data in the study. 

**Genomic analysis**

During genomic analysis, investigators were blinded to clinical metadata. All samples were sequenced using either Illumina or Oxford Nanopore Technology, depending on availability at the time of sequencing. For Illumina sequencing, raw data were processed with the ARTIC NextFlow pipeline (https://github.com/connorlab/ncov2019-artic-nf/tree/illumina, last updated April 2020).  Consensus sequences were called at 10X minimum coverage across the genome. For Nanopore sequencing, raw data in the form of fast5 files were base called using the ONT guppy high accuracy basecaller included in ONT MinkNOW for the GridION version 19.12.6 (Oxford Nanopore Technologies, Oxford, UK).. Fastq files produced were demultiplexed using Porechop under the artic-ncov2019 version 1.0 pipeline (https://github.com/artic-network/artic-ncov2019). Consensus sequences at a minimum of 20X coverage were generated using the artic-ncov2019 medaka pipeline (https://artic.network/ncov-2019/ncov2019-bioinformatics-sop.html). Only samples with > 50% genome coverage were carried forward for genomic analysis.

A maximum likelihood tree of the consensus alignment was constructed using IQ-TREE v.2.1.2, with the GTR model and 1000 bootstrap replicates[6]. Trees were rooted on the SARS-CoV-2 reference genome MN908947.3, and visualized with ggtree. Consensus sequences were aligned using MAFFT[7]. Figures were generated in R 3.6.1 using Rstudio 1.2 with libraries dplyr [https://CRAN.R-project.org/package=dplyr], ggplot2 [ggplot2: Elegant Graphics for Data Analysis. Springer-Verlag New York, 2016] and ggtree ([http://tree.bio.ed.ac.uk/software/figtree/](http://tree.bio.ed.ac.uk/software/figtree/))[8, 9]. Samples with >90% coverage and 10X depth were carried forward for analysis. Minority variant alleles (MVAs) were called from Illumina sequenced samples with >90% genome coverage. MVAs were called relative to Wuhan-1 (MN908947.3), with a frequency of above 5% and with a minimum of 4 supporting reads identified at sites with a read depth of ≥ 5 using VarScan[10].

**References**

1.	Ong SWX, Young BE, Lye DC. Lack of detail in population-level data impedes analysis of SARS-CoV-2 variants of concern and clinical outcomes. The Lancet Infectious Diseases.

2.	Hennessy S, Bilker WB, Berlin JA, Strom BL. Factors influencing the optimal control-to-case ratio in matched case-control studies. Am J Epidemiol **1999**; 149(2): 195-7.

3.	Grant PR, Turner MA, Shin GY, Nastouli E, Levett LJ. Extraction-free COVID-19 (SARS-CoV-2) diagnosis by RT-PCR to increase capacity for national testing programmes during a pandemic. bioRxiv **2020**: 2020.04.06.028316.

4.	Public Health England. The Green Book, Chapter 6: Contraindications and special considerations. Public Health England **2017** [**https://assets.publishing.service.gov.uk/government/uploads/system/uploads/attachment_data/file/655225/Greenbook_chapter_6.pdf**](https://assets.publishing.service.gov.uk/government/uploads/system/uploads/attachment_data/file/655225/Greenbook_chapter_6.pdf).

5.	Harder VS, Stuart EA, Anthony JC. Propensity score techniques and the assessment of measured covariate balance to test causal associations in psychological research. Psychol Methods **2010**; 15(3): 234-49.

6.	Minh BQ, Schmidt HA, Chernomor O, et al. IQ-TREE 2: New Models and Efficient Methods for Phylogenetic Inference in the Genomic Era. Mol Biol Evol **2020**; 37(5): 1530-4.

7.	Katoh K, Standley DM. MAFFT Multiple Sequence Alignment Software Version 7: Improvements in Performance and Usability. Molecular Biology and Evolution **2013**; 30(4): 772-80.

8.	Racine JS. RStudio: A Platform-Independent IDE for R and Sweave. Journal of Applied Econometrics **2012**; 27(1): 167-72.

9.	Yu G. Using ggtree to Visualize Data on Tree-Like Structures. Current Protocols in Bioinformatics **2020**; 69(1): e96.

10.	Koboldt DC, Zhang Q, Larson DE, et al. VarScan 2: Somatic mutation and copy number alteration discovery in cancer by exome sequencing. Genome Research **2012**; 22(3): 568-76.
